# Rational engineering of a native hyperthermostable lactonase into a broad spectrum phosphotriesterase

**DOI:** 10.1038/s41598-017-16841-0

**Published:** 2017-12-01

**Authors:** Pauline Jacquet, Julien Hiblot, David Daudé, Céline Bergonzi, Guillaume Gotthard, Nicholas Armstrong, Eric Chabrière, Mikael Elias

**Affiliations:** 1 CNRS UMR 7278, IRD198, INSERM U1095, APHM, Institut Hospitalier Universitaire Méditerranée-Infection, Aix-Marseille Université, 19-21 Bd Jean Moulin, 13005 Marseille, France; 2 Gene&GreenTK, IHU Méditerranée Infection, 19-21 Bd Jean Moulin, 13005 Marseille, France; 3 University of Minnesota, Department of Biochemistry, Molecular Biology and Biophysics & Biotechnology Institute, St. Paul, MN 55108 USA; 4 Present Address: MPI for Medical Research, Chemical Biology department (EPFL), Heidelberg, Germany

## Abstract

The redesign of enzyme active sites to alter their function or specificity is a difficult yet appealing challenge. Here we used a structure-based design approach to engineer the lactonase *Sso*Pox from *Sulfolobus solfataricus* into a phosphotriesterase. The five best variants were characterized and their structure was solved. The most active variant, αsD6 (V27A-Y97W-L228M-W263M) demonstrates a large increase in catalytic efficiencies over the wild-type enzyme, with increases of 2,210-fold, 163-fold, 58-fold, 16-fold against methyl-parathion, malathion, ethyl-paraoxon, and methyl-paraoxon, respectively. Interestingly, the best mutants are also capable of degrading fensulfothion, which is reported to be an inhibitor for the wild-type enzyme, as well as others that are not substrates of the starting template or previously reported W263 mutants. The broad specificity of these engineered variants makes them promising candidates for the bioremediation of organophosphorus compounds. Analysis of their structures reveals that the increase in activity mainly occurs through the destabilization of the active site loop involved in substrate binding, and it has been observed that the level of disorder correlates with the width of the enzyme specificity spectrum. This finding supports the idea that active site conformational flexibility is essential to the acquisition of broader substrate specificity.

## Introduction

Enzymes are extraordinary molecules that can accelerate chemical reactions, including those that spontaneously occur extremely slowly, and can make them happen many times per second^1^. Some of these reactions are of high biotechnological or industrial interest and enzymes are, therefore, the focus of numerous studies^2^. However, enzymes are often not directly compatible with industrial processes for various reasons ranging from their sub-optimality^3,4^ or lack of stability under certain conditions^5^. The properties of enzymes can be tuned by molecular engineering to fit these new requirements using high throughput methods such as directed evolution^5–10^ and /or low throughput methods, reducing the size of the library by focusing on positions that can be saturated^11–13^, or by using phylogenetically-inferred^14,15^ or structure-based mutations to create combinatorial^16^, reduced libraries^17^.

Here, we focus on the molecular engineering of an enzymatic bio-decontaminant of organophosphates compounds (OPs), including nerve agents and insecticides. These compounds are neurotoxic because of their ability to irreversibly inhibit the acetylcholine esterase, a key enzyme in the nervous system^18^. Massively used in agriculture as pesticides^19^, they were modified before World War II to increase their toxicity and develop chemical warfare agents^20^. OPs still represent major environmental, health and security concerns, as they cause significant pollution^21,22^, and intoxication, and have been used in terrorist attacks^19,20^. However, since current methods for removing them are cost prohibitive and lead to ecological concerns, enzymatic remediation is a promising way of developing efficient bio-decontamination methods^23–25^.

Phosphotriesterase-like Lactonases (PLLs) are appealing candidates, being natural lactonases endowed with promiscuous phosphotriesterase activity^26^. They have long been related to the bacterial phosphotriesterases (PTEs), enzymes which are capable of hydrolyzing insecticides such as parathion with high catalytic efficiency^27^. PTEs and PLLs belong to the amidohydrolase superfamily^28,29^, and share the same (β/α)_8_ barrel topology. Some PLL representatives are extremely thermostable^30–38^ and are particularly promising candidates for biotechnological use^39,40^ by virtue of their high stability and resistance towards denaturing agents^41^. PLLs, however, exhibit much lower phosphotriesterase activity than PTEs. Engineering experiments with the aim of increasing PLLs’ phosphotriesterase activity were performed. In particular, because PTEs and PLLs’ active sites mainly differ in their loop length (7 and 8), sequences and conformations^16,26,42^, loop grafting experiments were performed and proved to be difficult. This approach resulted in aggregation-prone or inactive enzymes^16,34^, but was successful when using a stepwise approach^43^. Recently, more classical engineering techniques were successful in increasing the phosphotriesterase activity of the PLL *Dr*OPH^44^.

In this study, we worked on the PLL *Sso*Pox from the hyperthermophilic archaea *Sulfolobus solfataricus* ^32,45^. *Sso*Pox exhibits high acyl-homoserine lactones and oxo-lactones hydrolysis abilities^11^, and promiscuous, low phosphotriesterase activity^46^. Previous work showed that engineering can successfully improve *Sso*Pox catalytic efficiency against phosphotriesters such as ethyl-paraoxon^11,47^. These previous studies highlighted the role of a key active site loop 8 residue, W263, which modulates the active site loop conformational space^11^. In this study, we aimed to further improve the phosphotriesterase activity of *Sso*Pox, including against phosphothionoesters. *Sso*Pox is a very appealing candidate for the bioremediation of phosphotriesters because of its unique thermal stability, and its ability to resist aging, solvent and protease treatments^48^. On the other hand, *Sso*Pox is a poor phosphothionoesterase, exhibiting a strong preference (100-fold) for methyl-paraoxon over methyl-parathion, a feature referred to as the thiono-effect^46^. We used a rational strategy: taking advantage of the structural similarity between *Sso*Pox and the bacterial PTE from the mesophilic *Brevundimonas diminuta* (*Bd*PTE)^27^, we designed and produced a structure-based combinatorial library, and screened this library for improved phosphotriesterase activity with the aim of obtaining heat stable, highly active variants with broad specificity. We obtained several improved variants, including against methyl-parathion, with a 2,210-fold improvement in activity. All these variants but variant αsB5 contained a mutation at position 263. Interestingly, the addition of the W263 mutation using site-saturation mutagenesis^12^ on an αsB5 background revealed an incompatibility of these mutations, instead of the expected increase in phosphotrieseterase activity. Of these improved variants, we obtained a mutant that lost the thiono-effect against methyl-parathion, exhibiting a >2,000-fold (k_cat_/K_M_ ~10^4^ M^−1^.s^−1^) as referred to the wild-type enzyme, and a methyl-paraoxon/methyl-parathion activity ratio of ~1. The X-ray structures of several improved variants reveal that the selected mutations increase the active site loop conformational flexibility and reshape the active site. The best variants were extensively characterized against eight commercially available insecticides, along with previously reported *Sso*Pox monovariants harboring mutation at residue 263 and their specificity spectrum was determined.

## Results and Discussion

**Figure 1:**
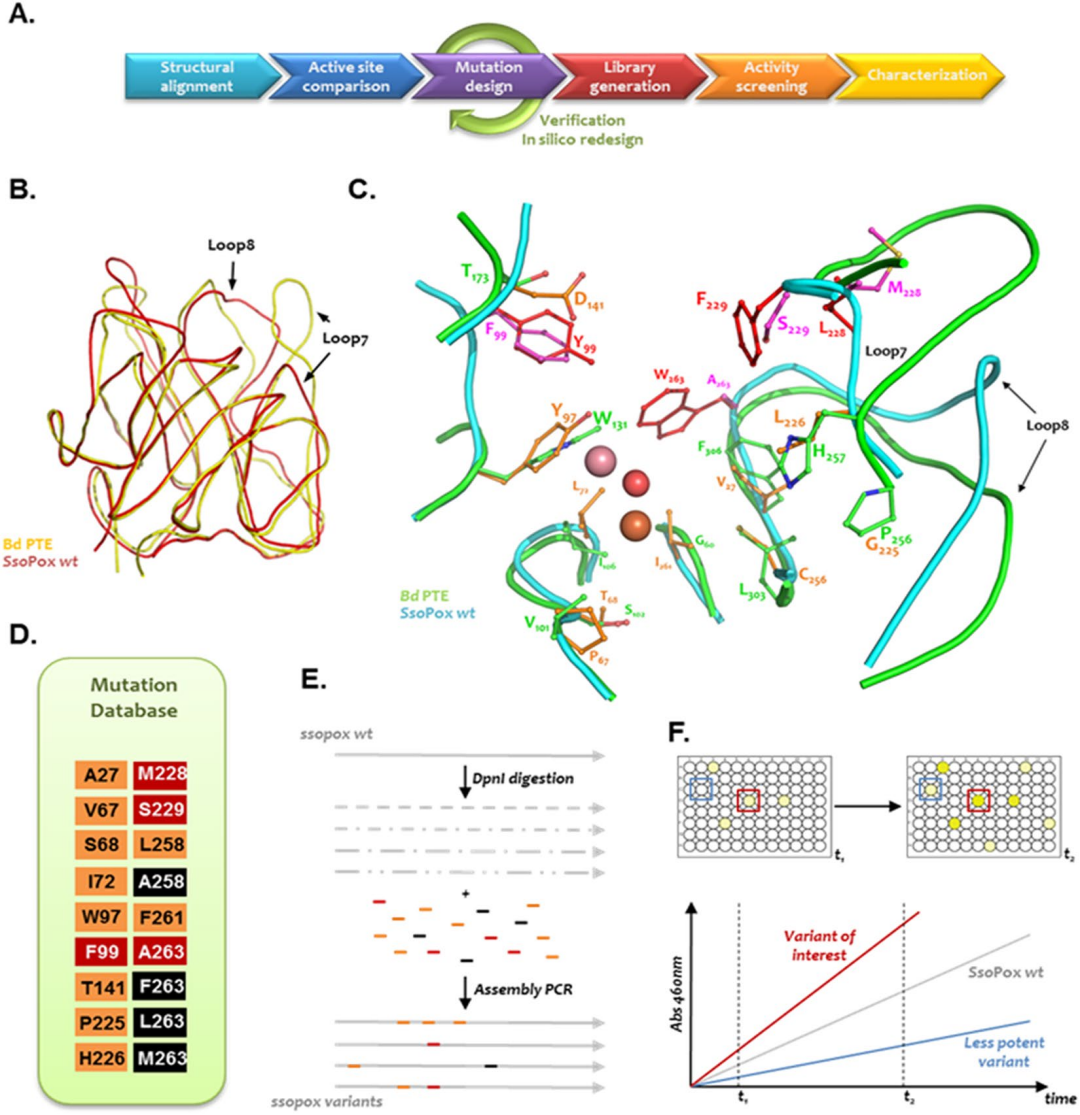
Structure-based design approach. (**A**) Pipeline used for the selection of improved mutants. (**B**) Structural alignments of *Sso*Pox (red) and *Bd*PTE structures (yellow) guided the mutational design. (**C**) Active site superposition of *Bd*PTE (green) and *Sso*Pox (cyan) active sites. Residues with structural equivalent (orange sticks) in both structures were used to design mutations of *Sso*Pox’s residues into the *Bd*PTE corresponding residues. Positions with no structural equivalent (red sticks) were designed using modelling tools (purple sticks) to mimic the active site cavity size, shape and chemical properties of the *Bd*PTE crystal structure model (Figure S1). (**D**) The mutations data base consists of mutations to the BdPTE sequence when there is a structural equivalence (orange), and of mutations designed when there is no structural equivalence (red). The third set of mutations (black) adds more diversity at two selected positions, 258 and 263. (**E**) Once the library had been validated, primers carrying mutations were used to shuffle them and generate a gene library with random combinations of selected mutations. (**F**) The library was screened for paraoxonase activity to identify enzymes with improved proficiency.

The lactonase *Sso*Pox was engineered for higher phosphotriesterase activity using structure-based combinatorial libraries. By comparing structures of enzymes with similar topology, it has been possible to redesign, using modelling tools, the active site cavity of *Sso*Pox to mimic as closely as possible that of *Bd*PTE (Fig. 1; Table S1). This method consisted of two main steps: (i) the identification of mutations corresponding to equivalent positions at the enzyme active sites using structural alignment and (ii) the rational selection of mutations, in a position with no equivalent residue, which mimicked the shape and chemical nature of the target enzyme cavity. A mutation dataset was thus obtained and used to develop a combinatorial library consisting of random combinations of our pre-selected mutations.

### Structure-based design produced mutants with improved phosphotriesterase activity

The structure-based design considerably reshaped the active site cavity of *Sso*Pox, theoretically bringing it closer to that of *Bd*PTE, both in shape and nature (Figure S1). A total of 14 key positions within the active site were identified, and were mutated to specific residues, or degenerated into several possible amino acids (see Methods). The paraoxonase activity of 184 randomly selected clones was screened and the 14 best were sequenced. Most of these 14 were found to possess the same mutations (e.g. W263F/L/M/A; 73% of cases) and (Figure S2A; Table S3). Of them, the most active variants, αsA1 (C258L-I261F-W263A), αsA6 (F46L-C258A-W263M-I280T), αsB5 (V27A-I76T-Y97W-Y99F-L130P-L226V), αsC6 (L72I-Y99F-I122L-L228M-F229S-W263L), and αsD6 (V27A-Y97W-L228M-W263M) were subject to kinetic and structural characterization. It should be noted that αsA1 was obtained in a previous engineering effort^47^, and that four of the five selected variants contained a substitution of the key residue W263. We previously highlighted the role of this residue in substrate binding and modulation of the active site loop 8 conformation, resulting in an increase in activity against promiscuous substrates^11^. Variant αsB5 does not harbor any substitution at position W263. We therefore decided to test the W263 substitution on an αsB5 background, using saturation mutagenesis strategy.

**Figure 2:**
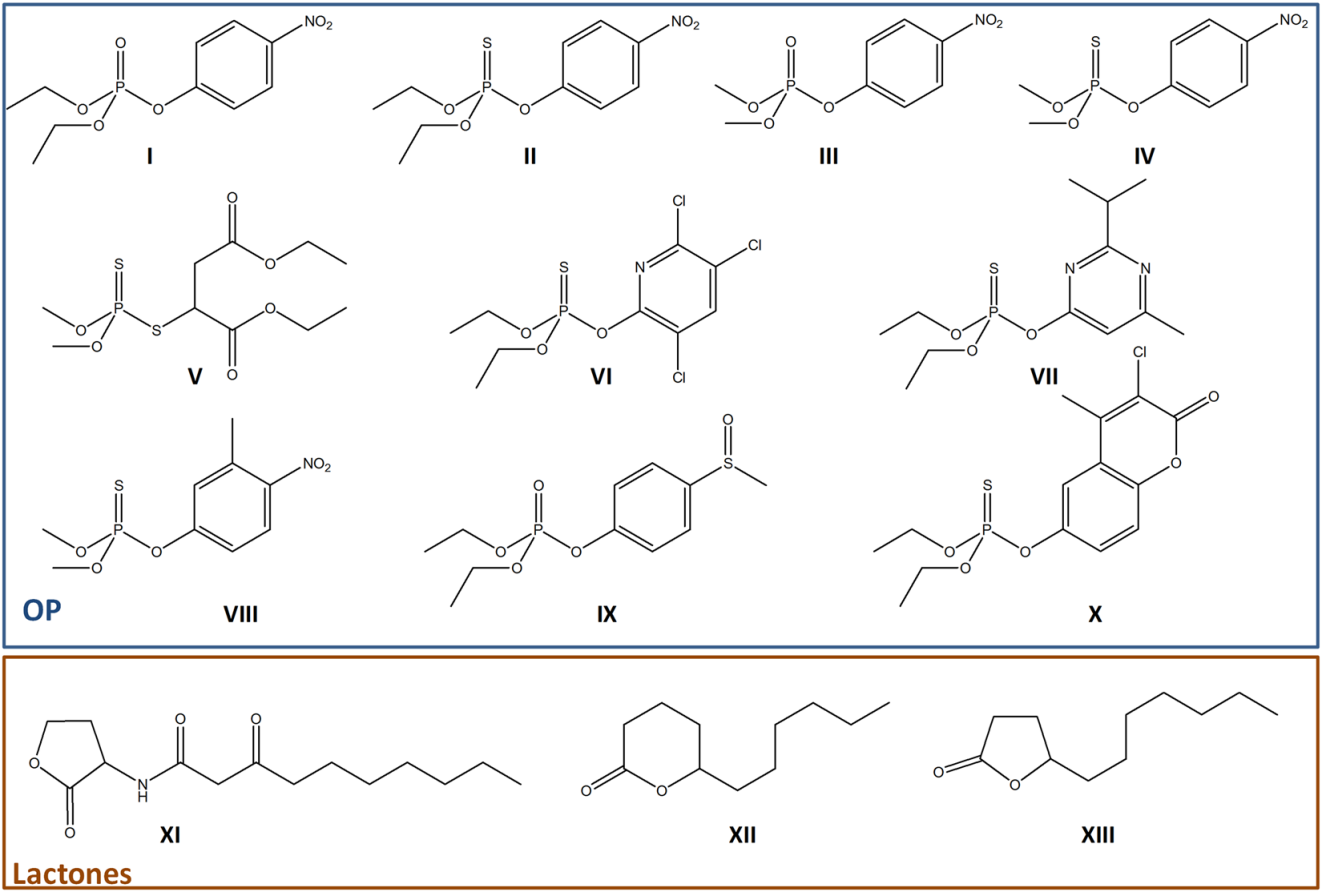
Chemical structure of substrates used in this study. The chemical structure of ethyl-paraoxon (**I**), ethyl-parathion (**II**), methyl-paraoxon (**III**), methyl-parathion (**IV**), malathion (**V**), chlorpyrifos (**VI**), diazinon (**VII**), fenitrothion (**VIII**), fensulfothion (**IX**), coumaphos (**X**), 3-oxo-C10 AHL (*l*) (**XI**), undecanoic-γ-lactone (**XII**) and undecanoic-δ-lactone (X**III**) generated using ChemDraw software.

**Figure 3:**
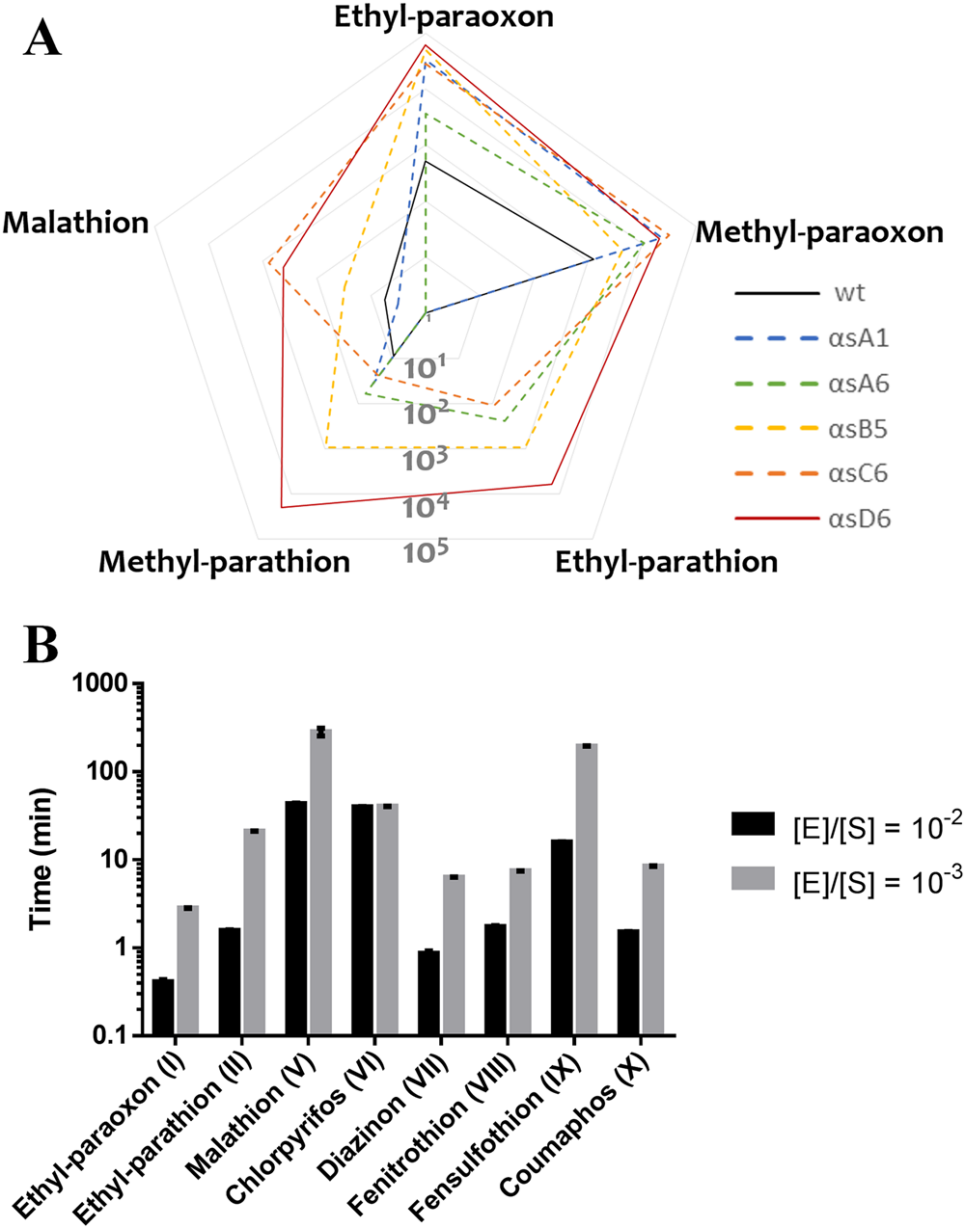
Activity characterization of the improved variants and bioremediation potential. (**A)** Phosphotriesterase catalytic efficiency comparison between wt-*Sso*Pox and the best selected variants. Data for wt-*Sso*Pox are from Hiblot *et al*.^46^. (**B)** Determination of the reaction time for hydrolyzing 95% using the αsD6 variant of a solution of pesticide at 250 µM. Two enzyme-to-substrate ratios ($\text{[E]/}[{\rm{S}}]$), 10^−2^ and 10^−3^, were considered.

The five variants (αsA1, αsA6, αsB5, αsC6 & αsD6) were characterized against several phosphotriesters, including ethyl-paraoxon (**I**), ethyl-parathion (**II**), methyl-paraoxon (**III**), methyl-parathion (**IV**) and malathion (**V**) (Fig. 2). Overall, all variant exhibits improved catalytic efficiencies against all tested substrates, with the exception of αsA1 with malathion (~2-fold decrease) (Fig. 3A; Table 2). The best improved variant, αsD6, exhibits a 2,210-fold increase in catalytic efficiency against methyl-parathion (Table 2). The largest improvements were observed for ethyl/methyl-parathion, two bad substrates for wild-type *Sso*Pox. Interestingly, while the *wt* enzyme shows a clear preference for small substituents^46^, most selected variants lost this preference, possibly indicating an enlargement of the active site cavity. These improvements were in the range of what was previously obtained but with higher intensive mutation protocols and for only one OP substrate^49^. Finally, it was noted that two variants (αsA6 & αsC6) presented a substrate inhibition for some thiono-phosphotriesters.

**Table 2:** Kinetic characterization of *Sso*Pox variants.

**Substrate**	***Sso*** **Pox**	**k**_**cat**_ **(s** ^−1^ **)**	**K**_**M**_ **(µM)**	**K**_**I**_ **(µM)**	**k**_**cat**_ **/K**_**M**_ **(M** ^−1^ **.s** ^−1^ **)**	**Enhancement/wt**
**Ethyl-paraoxon (I)**	wt°	1.26 ± 0.13 × 10^1^	2.43 ± 0.37 × 10^4^	—	5.12 ± 1.31 × 10^2^	1
	αsA1	2.70 ± 0.23 × 10^1^	8.00 ± 1.55 × 10^2^	—	3.37 ± 0.71 × 10^4^	65
	αsA6	5.87 ± 0.47	1.63 ± 0.63 × 10^3^	—	3.61 ± 1.43 × 10^3^	7
	αsB5	2.06 ± 0.14 × 10^1^	6.01 ± 0.96 × 10^2^	—	3.43 ± 1.46 × 10^4^	67
	αsC6	4.27 ± 0.25 × 10^1^	1.50 ± 0.16 × 10^3^	—	2.86 ± 0.35 × 10^4^	55
	αsD6	3.22 ± 0.18 × 10^1^	1.09 ± 0.12 × 10^3^	—	2.95 ± 1.55 × 10^4^	58
	αsB5 W263M	*	*	—	5.62 ± 0.11 × 10^3^	11
	αsB5 W263L	*	*	—	8.07 ± 0.28 × 10^3^	16
	αsB5 W263I	4.09 ± 0.89 × 10^1^	6.64 ± 1.75 × 10^3^	—	6.16 ± 5.07 × 10^3^	12
	W263L^+^	*	*	—	2.37 ± 0.33 × 10^3^	5
	W263M^+^	6.82 ± 0.57	9.31 ± 1.63 × 10^2^	—	7.33 ± 1.42 × 10^3^	14
**Ethyl-parathion (II)**	wt°	ND	ND	ND	ND	ND
	αsA1	ND	ND	ND	ND	ND
	αsA6	3.20 ± 0.20 × 10^−2^	1.34 ± 0.18 × 10^2^	3 250 ± 1020	2.39 ± 0.35 × 10^2^	ND
	αsB5	2.69 ± 0.15 × 10^−1^	2.72 ± 0.49 × 10^2^	—	9.86 ± 3.14 × 10^2^	ND
	αsC6	8.53 ± 0.30 × 10^−3^	7.70 ± 1.10 × 10^1^	—	1.10 ± 0.16 × 10^2^	ND
	αsD6	7.35 ± 0.36 × 10^−1^	5.34 ± 0.62 × 10^2^	—	1.38 ± 0.57 × 10^3^	ND
	αsB5 W263M	5.24 ± 0.23 × 10^−2^	2.02 ± 0.31 × 10^2^	—	2.59 ± 0.74 × 10^2^	ND
	αsB5 W263L	4.35 ± 0.16 × 10^−2^	3.46 ± 0.37 × 10^2^	—	1.26 ± 0.44 × 10^2^	ND
	αsB5 W263I	4.74 ± 0.21 × 10^−2^	2.61 ± 0.37 × 10^2^	—	1.82 ± 0.57 × 10^2^	ND
	W263L	ND	ND	—	ND	ND
	W263M	ND	ND	—	ND	ND
**Methyl-paraoxon (III)**	wt°	2.71 ± 0.64	2.14 ± 0.68 × 10^3^	—	1.27 ± 0.70 × 10^3^	1
	αsA1	4.35 ± 0.87 × 10^1^	1.90 ± 0.52 × 10^3^	—	2.29 ± 0.78 × 10^4^	18
	αsA6	5.89 ± 0.55	5.46 ± 1.02 × 10^2^	—	1.08 ± 0.23 × 10^4^	8
	αsB5	*	*	—	4.31 ± 0.14 × 10^3^	3
	αsC6	2.36 ± 0.37 × 10^1^	7.59 ± 2.04 × 10^2^	—	3.11 ± 0.97 × 10^4^	25
	αsD6	4.25 ± 0.52 × 10^1^	2.08 ± 0.34 × 10^3^	—	2.04 ± 0.42 × 10^4^	16
**Methyl-parathion (IV)**	wt°	1.10 ± 0.02 × 10^−3^	1.21 ± 0.10 × 10^2^	—	9.09 ± 0.90	1
	αsA1	1.30 ± 0.50 × 10^−2^	3.53 ± 0.34 × 10^2^	—	3.68 ± 1.46 × 10^1^	4
	αsA6	1.55 ± 0.15 × 10^−2^	2.54 ± 0.39 × 10^2^	1 520 ± 384	6.10 ± 1.11 × 10^1^	7
	αsB5	1.50 ± 0.04 × 10^−1^	1.58 ± 0.15 × 10^2^	—	9.49 ± 0.94 × 10^2^	104
	αsC6	3.00 ± 0.10 × 10^−3^	1.21 ± 0.15 × 10^2^	—	2.48 ± 0.32 × 10^1^	3
	αsD6	6.89 ± 0.35	3.43 ± 0.44 × 10^2^	—	2.01 ± 0.28 × 10^4^	2210
**Malathion (V)**	wt°	8.90 ± 0.40 × 10^−4^	1.60 ± 0.29 × 10^2^	—	5.56 ± 1.26	1
	αsA1	7.10 ± 0.40 × 10^−4^	2.22 ± 0.35 × 10^2^	—	3.20 ± 0.54	0.5
	αsA6	ND	ND	ND	ND	ND
	αsB5	1.84 ± 0.16 × 10^−2^	9.13 ± 1.61 × 10^2^	—	2.02 ± 1.01 × 10^1^	4
	αsC6	2.47 ± 0.44 × 10^−2^	3.20 ± 1.90 × 10^1^	820 ± 343	7.72 ± 4.78 × 10^2^	139
	αsD6	1.00 ± 0.02 × 10^−1^	1.13 ± 0.10 × 10^2^	—	9.08 ± 2.00 × 10^2^	163
	αsB5 W263M	8.92 ± 0.67 × 10^−3^	8.61 ± 1.32 × 10^2^	—	1.04 ± 0.51 × 10^1^	2
	αsB5 W263L	1.11 ± 0.08 × 10^−2^	9.28 ± 1.32 × 10^2^	—	1.20 ± 0.60 × 10^1^	2
	αsB5 W263I	1.18 ± 0.08 × 10^−2^	7.80 ± 1.08 × 10^2^	—	1.51 ± 0.71 × 10^1^	3
	W263L	ND	ND	—	ND	ND
	W263M	ND	ND	—	ND	ND
**Chlorpyrifos (VI)**	wt	ND	ND	—	ND	ND
	αsD6	*	*	—	3.12 ± 0.09 × 10^2^	ND
	αsB5	*	*	—	2.07 ± 0.05 × 10^2^	ND
	αsB5 W263M	*	*	—	1.64 ± 0.03 × 10^2^	ND
	αsB5 W263L	*	*	—	1.46 ± 0.03 × 10^2^	ND
	αsB5 W263I	*	*	—	1.42 ± 0.03 × 10^2^	ND
	W263L	ND	ND	—	ND	ND
	W263M	ND	ND	—	ND	ND
**Diazinon (VII)**	wt	ND	ND	—	ND	ND
	αsD6	8.93 ± 0.79	2.30 ± 0.31 × 10^3^	—	3.89 ± 2.56 × 10^3^	ND
	αsB5	1.26 ± 0.06	4.10 ± 0.65 × 10^2^	—	3.03 ± 0.98 × 10^3^	ND
	αsB5 W263M	3.89 ± 0.38 × 10^−1^	3.91 ± 1.04 × 10^2^	—	9.92 ± 3.65 × 10^2^	ND
	αsB5 W263L	5.61 ± 0.23 × 10^−1^	4.74 ± 0.53 × 10^2^	—	1.18 ± 0.44 × 10^3^	ND
	αsB5 W263I	4.01 ± 0.30 × 10^−1^	3.24 ± 0.72 × 10^2^	—	1.24 ± 0.42 × 10^3^	ND
	W263L	ND	ND	—	ND	ND
	W263M	ND	ND	—	ND	ND
**Fenitrothion (VIII)**	wt	ND	ND	—	ND	ND
	αsD6	7.94 ± 0.50 × 10^−1^	5.28 ± 0.80 × 10^2^	—	1.50 ± 0.62 × 10^3^	ND
	αsB5	1.40 ± 0.20 × 10^−2^	3.14 ± 1.43 × 10^2^	—	9.11 ± 1.40 × 10^1^	ND
	αsB5 W263M	2.22 ± 0.12 × 10^−2^	3.31 ± 0.53 × 10^2^	—	6.69 ± 2.31 × 10^1^	ND
	αsB5 W263L	2.63 ± 0.13 × 10^−2^	6.54 ± 0.75 × 10^2^	—	4.01 ± 1.79 × 10^1^	ND
	αsB5 W263I	2.01 ± 0.08 × 10^−2^	3.97 ± 0.46 × 10^2^	—	5.05 ± 1.81 × 10^1^	ND
	W263L	3.66 ± 0.15 × 10^−3^	4.15 ± 0.44 × 10^2^	—	8.82 ± 3.32	ND
	W263M	9.49 ± 0.43 × 10^−4^	1.57 ± 0.28 × 10^2^	—	6.06 ± 1.55	ND
**Fensulfothion (IX)**	wt	ND	ND	—	ND	ND
	αsB5	4.28 ± 0.39 × 10^−2^	9.41 ± 1.72 × 10^2^	—	4.55 ± 2.30 × 10^1^	ND
	αsD6	4.06 ± 1.60 × 10^−1^	8.60 ± 3.97 × 10^3^	—	4.72 ± 4.04 × 10^1^	ND
	αsB5 W263M	*	*	—	1.12 ± 0.46 × 10^1^	ND
	αsB5 W263L	4.94 ± 1.35 × 10^−2^	2.57 ± 1.04 × 10^3^	—	1.92 ± 1.30 × 10^1^	ND
	αsB5 W263I	*	*	—	6.50 ± 0.07	ND
	W263L	ND	ND	—	ND	ND
	W263M	ND	ND	—	ND	ND
**Coumaphos (X)**	wt	ND	ND	—	ND	ND
	αsD6	*	*	—	1.64 ± 0.02 × 10^4^	ND
	αsB5	*	*	—	7.82 ± 0.14 × 10^3^	ND
	αsB5 W263M	*	*	—	1.15 ± 0.02 × 10^3^	ND
	αsB5 W263L	*	*	—	1.16 ± 0.05 × 10^3^	ND
	αsB5 W263I	*	*	—	1.11 ± 0.04 × 10^3^	ND
	W263L	*	*	—	6.01 ± 0.18	ND
	W263M	*	*	—	4.80 ± 0.32	ND
**3-oxo-C10-AHL (** ***l*** **) (XI)**	wt°	*	*	—	3.16 × 10^4^	1
	αsA1	*	*	—	1.41 × 10^2^	0.004
	αsA6	*	*	—	1.78 × 10^3^	0.06
	αsB5	ND	ND	—	ND	ND
	αsC6	*	*	—	5.84 × 10^2^	0.02
	αsD6	ND	ND	—	ND	ND
**Undecanoic-γ-lactone (XII)**	wt°	*	*	—	2.36 × 10^3^	1
	αsA1	*	*	—	1.63 × 10^3^	0.7
	αsA6	*	*	—	4.40 × 10^5^	186
	αsB5	*	*	—	9.05 × 10^2^	0.4
	αsC6	*	*	—	5.73 × 10^5^	243
	αsD6	*	*	—	1.60 × 10^4^	7
**Undecanoic-δ-lactone (XIII)**	wt°	*	*	—	7.86 × 10^4^	1
	αsA1	*	*	—	2.55 × 10^3^	0.03
	αsA6	*	*	—	9.65 × 10^6^	123
	αsB5	*	*	—	5.33 × 10^3^	0.08
	αsC6	*	*	—	3.60 × 10^5^	5
	αsD6	*	*	—	7.50 × 10^4^	0.9

*Corresponds to undetermined values because enzyme saturation could not be reached.

ND corresponds to undetectable values because substrates were not hydrolyzed or too poorly hydrolyzed to be observable.

°Data taken from Hiblot *et al*.^46^.

^+^Data taken from Hiblot *et al*.^11^.

The five selected variants were further evaluated for their lactonase activity (Table 2). Interestingly, variants αsA6 and αsC6 exhibit enhanced lactonase activity against γ- and δ-lactones, while αsA1, αsB5 and αsD6 show reduced lactonase catalytic efficiencies. This emphasizes the fact that the improvement of the phosphotriesterase activity does not necessarily compromise the cognate, lactonase activity of the enzyme. These mutations therefore dramatically increased a new activity (phosphotriesterase) without the complete loss of the native/original function (lactonase)^4^. These mutants are different from those generated on another PLLs, through loop insertion. While the stepwise insertion of residues in loop 7 can significantly increase the ability of PLLs to degrade phosphotriesters, it also drastically decreases their lactonase activity^43^. Insertion in loop 7 has been previously described as a key evolutionary event in the transition from lactonase to phosphotriesterase^50^.

### Structural analysis of improved variants reveals increased mobility of the active site loop 8

The crystal structures of variants αsA1, αsA6, αsB5, αsC6 and αsD6 were solved (Table 1). While most structures were solved at high or medium resolutions (1.4–2.55 Å), mutant asD6 could only be solved at low resolution (2.95 Å). Given this resolution, we limited our interpretations and retained this data for the consistency of the study as it exhibits structural features (loop 8 disorder) that are consistent with all the other mutants. Overall, the mutants’ structures are similar to that of *wt*-*Sso*Pox. However, the active site cavities are larger for the mutants: most selected mutations replace residues with smaller ones: W263 is mutated into M/L/I, V27 into A, I76 into T, L130 into P, Y99 into F. Only two selected substitutions relate to bulkier side chains: Y97W and L228M. This enlargement is difficult to quantify even with the structure of these mutants because of the extremely mobile nature of loop 8 in the variants. Enlarging the active site was a main objective of the active site redesign of *Sso*Pox, to enable the bulkier phosphotriesters to bind within the natural lactonase enzyme. Additionally, the loop 8 conformations differ in mutants, as compared to the *wt*-enzyme. Because part of loop 8, including position 263, is located at the enzyme dimer interface, altered loop 8 conformations modulate the relative orientation of both monomers. In the case of the mutants characterized in this study, the dimer reorientation yields significant displacement up to 5.2 Å (e.g., αsA1, between equivalent carbon α positions), as compared to *wt*-*Sso*Pox (Figure S5). Similar reorientations were previously observed upon substrate binding or mutations of W263^11,42^.

**Table 1:** Data collection and refinement statistics of *Sso*Pox variants structures.

Data collections	asA6 open	asA6 closed	asB5	asC6	asD6	asA1
PDB ID	5VRK	5VRI	5W3U	5W3Z	5W3W	5VSA
Wavelength (Å)	0.97934	0.93340	0.97625	1.00882	1.00882	0.99987
Resolution (Å)	1.4	2.15	2.5	2.55	2.95	2.0
Space group	P2_1_	P2_1_2_1_2_1_	P2_1_2_1_2_1_	P2_1_2_1_2_1_	P2_1_2_1_2_1_	P2_1_2_1_2_1_
**Unit cell dimensions**						
a (Å)	49.28	87.17	82.93	81.65	82.34	86.6
b (Å)	137.20	103.61	105.95	105.80	105.01	103.100
c (Å)	49.36	152.61	152.83	154.53	153.15	151.8
α (°)	90.0					
β (°)	98.7					
γ (°)	90.0					
No. observed reflections	419571 (75591)	408826 (24712)	206443 (23317)	194083 (10849)	106247 (5379)	703275 (96197)
No. unique reflections	121915 (22376)	74124 (4744)	47092 (5182)	43204 (2416)	28163 (1372)	91103 (12398)
Completeness (%)	96.1 (94.3)	97.8 (95.5)	99.5 (99.8)	97.3 (98.0)	98.3 (99.9)	98.6% (99.7%)
Rmerge (%)	8.2 (33.4)	9.8 (44.6)	6.1 (48.7)	5.55 (42.8)	6.6 (46.0)	9.0% (48.8%)
Rmeasure (%)	9.7 (39.5)	10.8 (49.3)	6.9 (55.4)	6.2 (48.5)	7.7 (52.9)	9.6% (52.6%)
I/σ(I)	9.07 (3.39)	14.31 (3.47)	12.84 (2.50)	15.68 (2.67)	12.89 (2.75)	15.59 (4.23)
Last resolution shell	1.5–1.4	2.2–2.15	2.6–2.5	2.6–2.55	3.0–2.95	2.1–2.0
Redundancy	3.44 (3.38)	5.51 (5.21)	4.38 (4.50)	4.49 (4.49)	3.77 (3.92)	7.72 (7.76)
**Refinement statistics**						
Resolution range (Å)	48.7128–1.40	49.0556–2.1500	49.6449–2.500	46.313–2.55	49.667–2.95	48.812–2.0
No. Reflections	115818	70416	44736	41042	26754	86547
Rfree /Rwork	16.84 / 13.36	21.35/17.23	27.17/21.22	24.15/19.97	28.31/24.68	20.55/16.32
No. protein atoms	5368	10122	9310	9657	9683	9987
No. water molecules	634	659	76	154	23	733
Average B factor (Å2)	19.095	24.984	86.6	80.375	95.992	29.885
**rmsd from ideal**						
Bond lengths (Å)	0.0088	0.0028	0.0050	0.0110	0.0070	0.0211
Bond angles (°)	1.0703	0.7309	0.8490	1.1730	0.9630	0.6950

**Figure 4:**
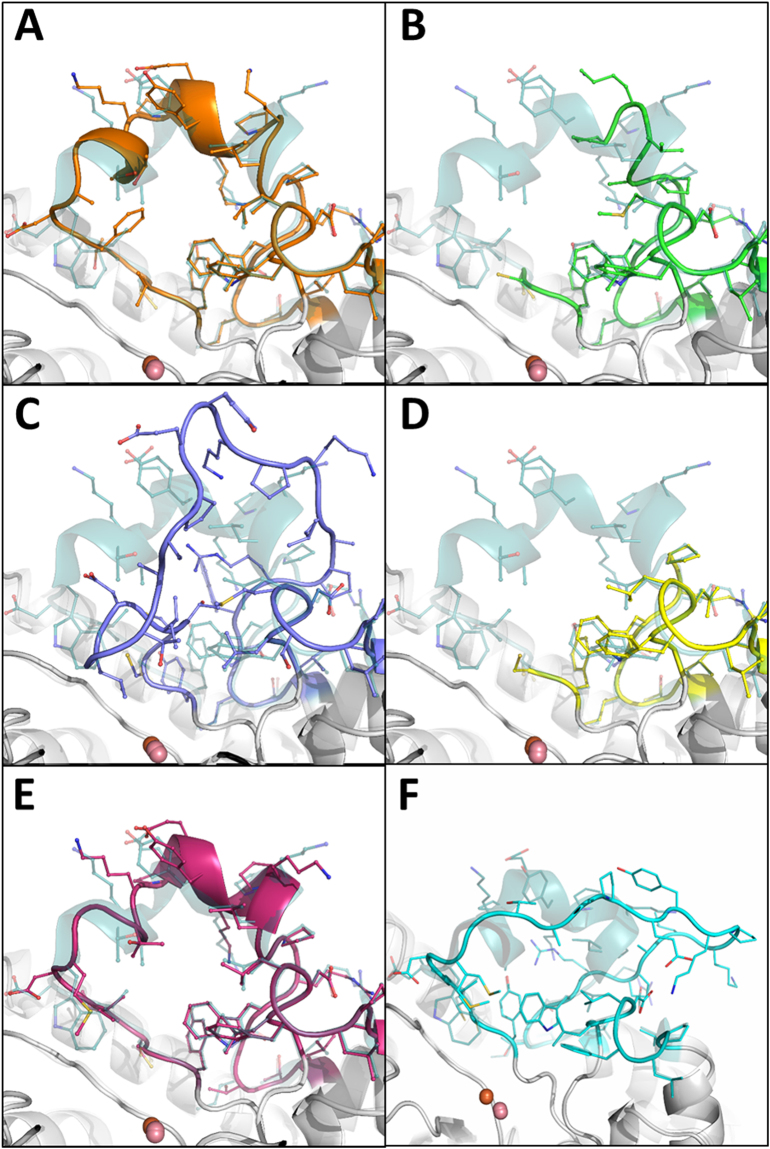
Loop 8 conformations in improved mutants. Active site loop 8 region of mutant structures superimposed with the wt-*Sso*Pox structure. The active site location is indicated by the presence of the bi-metallic catalytic center shown as spheres. The wt-*Sso*Pox structure loop 8 conformation is shown as dark green cartoon. (**A**) αsA1′s loop 8 is shown in orange cartoon, (**B**) αsD6′s loop 8 is shown in green cartoon, and is not entirely visible in the electronic density maps, (**C**) αsC6′s loop 8 is shown in blue cartoon, (**D**) αsB5′s loop 8 is shown in yellow cartoon, and is not entirely visible in the electronic density maps, (**E**) αsA6-CC’s loop 8 is shown in magenta cartoon, (**F**) αsA6-OC’s loop 8 is shown in cyan cartoon.

**Figure 5:**
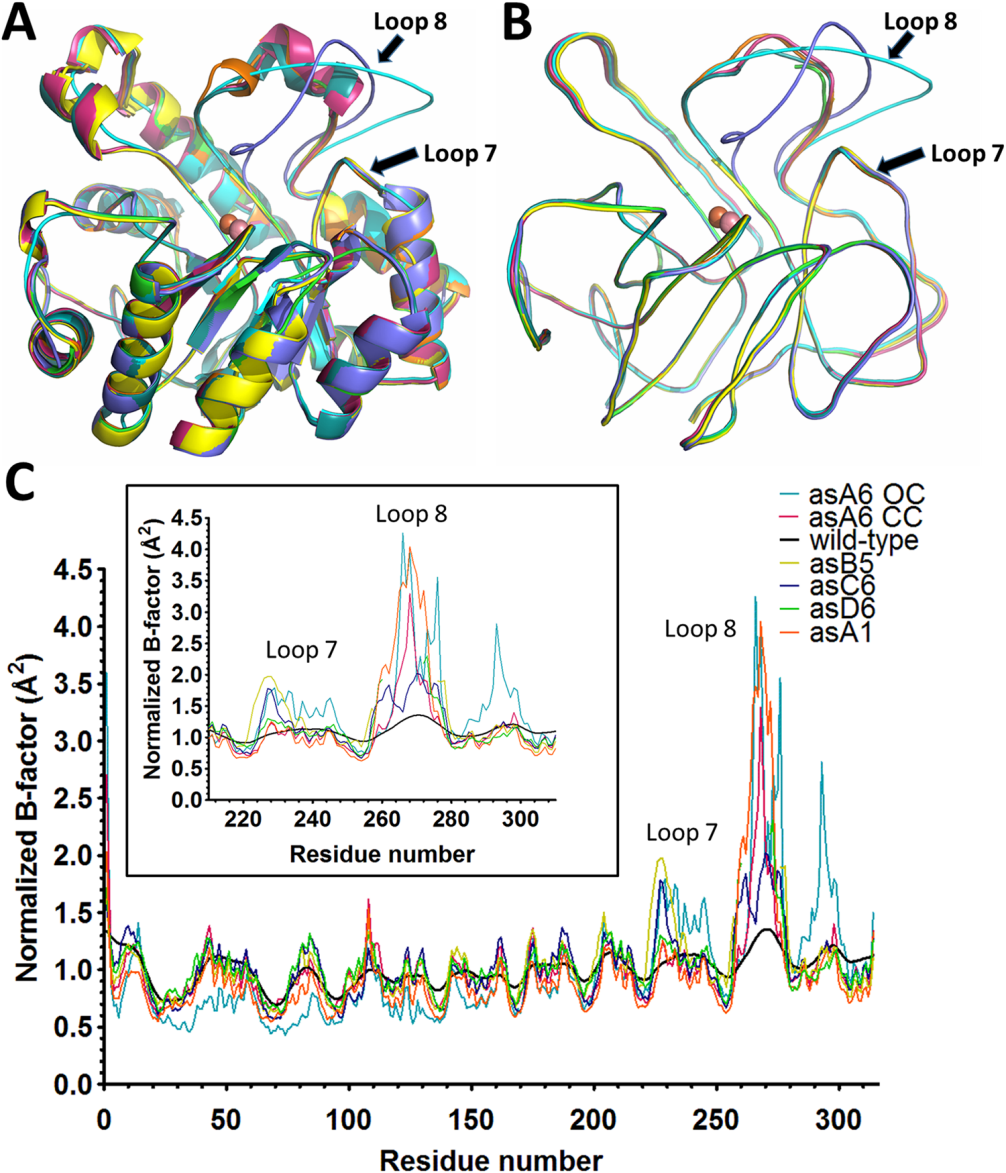
Mutants’ active site loop 8 exhibit higher conformational flexibility. (**A**) Cartoon representation of the superposition of the structures (monomers) of wt-*Sso*pox (dark green), αsA1 (orange), αsB5 (yellow), αsD6 (green), αsC6 (blue), αsA6-CC (magenta) and αsA6-OC (cyan). (**B**) Smoothed ribbon representation of the superposition of the structures (monomers) of of wt-*Sso*pox (green), αsA1 (orange), αsB5 (yellow), αsD6 (green), αsC6 (blue), αsA6-CC (magenta) and αsA6-OC (cyan). (**C**) Positional distributions of normalized B-factor values (the x-axis represents residue number) for wt-*Sso*Pox (black line), αsA1 (orange), αsB5 (yellow), αsD6 (green), αsC6 (blue), αsA6-OC (blue line) and (magenta line). A zoomed inset highlights the loop 7 and 8 sequence region.

Although the active sites of the improved variant structures superpose well onto the *wt*-structure, all selected mutants display altered loop 8 conformations. Changes are subtle for some of the variants (e.g. αsA1), but are large for others (e.g. αsC6) (Fig. 4). Notably, mutant αsA6 was crystallized in two different conformations: one in which loop 8 adopts a *wt*-like conformation, referred to as closed conformation (CC), and another one where loop 8 is unfolded, referred to as open conformation (OC) (Figure S6). In the two other mutants, αsD6 and αsB5, loop 8 could not be modeled due to the lack of electronic density, which was likely to be due to the high level of motion of this enzyme region. Analysis of the normalized thermal motion B-factor supports this hypothesis. It also confirms that loop 8 is highly mobile in all selected mutants, as compared to *wt*-*Sso*Pox (Fig. 5). The higher mobility of loop 8 may also partly explain the improved ability of the variants to hydrolyze phosphotriesters with large substituents.

### Improved mutant αsD6 lost the thiono-effect

**Figure 6:**
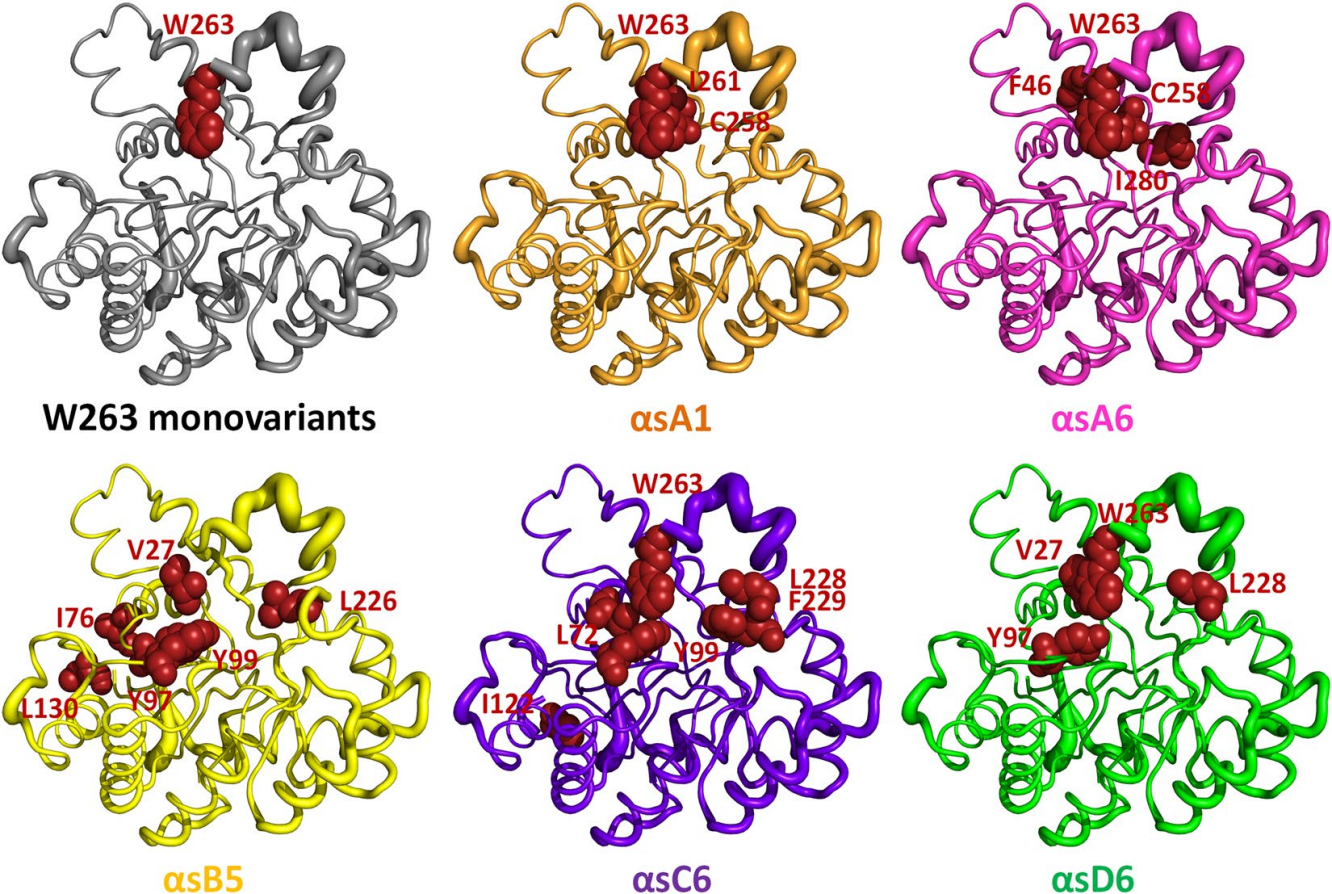
Localization of mutations harbored by *Sso*Pox monovariants, αsA1, αsA6, αsB5, αsC6 and αsD6. Mutations are highlighted in red spheres using the SsoPox wild-type (PDB 3UF9) as a template.

*Sso*Pox presents a marked preference for oxono-OP substrates as compared to thiono-ones (>100-fold in catalytic efficiency)^46^. Conversely, PTEs do not exhibit such a drastic preference, paraoxon being a slightly better substrate than parathion for the enzyme^27,51^. Interestingly, some selected variants retained a strong preference for oxono-phosphotriesters (~10-fold), whereas others such as αsD6 lost the thiono-effect (Figure S7). The wild-type enzyme exhibits a low K_M_ value with methyl-parathion (121 µM), but also a very low k_cat_ value (1.10 × 10^−3^ s^−1^) (Table 2). This may indicate a stronger interaction between the thiono moiety and the metal cations (low K_M_ value), which would subsequently impair catalysis (low k_cat_ values)^52^. Interestingly, the improved variants do not exhibit significant difference in K_M_ values for thiono-phosphotriesters, but show a dramatic increase in k_cat_ values, as compared to wild-type enzymes (e.g. k_cat_ value of 6.89 s^−1^ and 1.10 × 10^−3^ s^−1^, as compared to methyl-parathion for the αsD6 variant and wild-type enzymes, respectively). The two selected variants, namely αsB5 and αsD6, which lost the thiono-effect have only two mutations in common: V27A and Y97W. Both residues are located relatively close to the bi-metallic active site, yet do not interact directly with the metals (V27-Fe: 6.7 Å, Y97-Co: 4.3 Å; Fig. 6). Because other PLLs have been shown to exhibit high charge coupling between the β metal and the conserved tyrosine residue^33,53^, Y97 is a likely candidate for the thiono-effect, and will be examined in future studies.

### Mutations of W263 and αsB5 are incompatible

αsB5 (V27A-I76T-Y97W-Y99F-L130P-L226V) is the only selected mutant that does not contain a substitution of position W263. Because W263 substitutions were previously shown to be key for increasing *Sso*Pox’s phosphotriesterase activity, we used saturation mutagenesis to introduce W263 mutations to the background of αsB5^12^. The best selected mutants against paraoxon (Figure S2C) were found to be αsB5-W263I/L/M. Surprisingly, none of these mutants demonstrated an increase in phosphotriesterase activity in comparison with αsB5, but rather a decrease in activity (Table 2). This is intriguing because, taken individually, these mutations have been shown to greatly improve phosphotriesterase activity. For example, with ethyl-paraoxon as a substrate, W263M and αsB5 produce a catalytic efficiency improvement of 14 and 67-fold, respectively, while the combination αsB5-W263M increases activity only 11-fold (Table 2). This example of negative epistasis could be due to the mode of action of these mutations. Structures reveal that αsB5 mutations have a destabilizing effect on loop 8, as illustrated by the B factor analysis. The same was observed for W263M in a previous study^11^. Combining these two destabilizing sets of mutations may have harmed the active site integrity, as previously described with another enzyme as “conformational active site disorder”^54^, and in particular the necessary alignment and pre-organization of the catalytic residues. The destabilizing effect of these mutations can also be observed on the overall enzyme stability, with αsB5, W263M, αsB5-W263M exhibiting T_m_ values of 70.4 °C, 85.3 °C and 69.1 °C, respectively, as compared to 106 °C for the wt enzyme (Table S4). Here, the effects of the combined 5 mutations harbored by variant αsB5 together with the previously identified positive substitutions at residue W263 were not additive underlining that single beneficial mutations do not necessarily induce positive epistatic effects. Synergistic effects of mutations are often complex to predict and most engineering strategies usually lead to an optimization plateau difficult to overcome^55^.

### Improvement of engineered variants over W263 mutation

In a previous report, we noted that mutation of W263 into all the other 19 residues lead to an increase of the promiscuous paraoxonase activity^11^. Here we note that most of the improved variants (i.e. αsA1, αsA6, αsB5, αsC6 & αsD6) (i) exhibit higher paraoxonase activity than W263L/M, and (ii) foremost are capable of hydrolyzing phosphotriesters that are not substrates for the wild-type enzyme or W263 mutants, such as ethyl-parathion, chlorpyrifos or fensulfothion (Table 2). The activity spectrum of these engineered variants against the tested phosphotriesters is therefore much wider than the wild-type enzyme and W263 mutants.

### Relation between active site loop disorder and broad enzymatic specificity

The organophosphorus compound bioremediation potential of several mutants was investigated. In particular, the best variants, αsD6 and αsB5, alongside other improved variants αsB5-W263I/L/M, W263L/M and the *wt*-enzyme, were characterized for their phosphotriesterase activity with ethyl-paraoxon (**I**), ethyl-parathion (**II**), malathion (**V**), chlorpyrifos (**VI**), diazinon (**VII**), fenitrothion (**VIII**), fensulfothion (**IX**) and coumaphos (**X**) (Fig. 2). Interestingly, we found that most mutants were able to hydrolyze fensulfothion, previously reported as an inhibitor of the wild-type enzyme^46^. Fensulfothion was indeed previously shown to bind head-to-tail into the wild-type enzyme active side^46^, a non-productive binding mode. Changes in the active site loop 8 conformational ensemble of engineered mutants might have allowed for the productive binding of fensulfothion.

**Figure 7:**
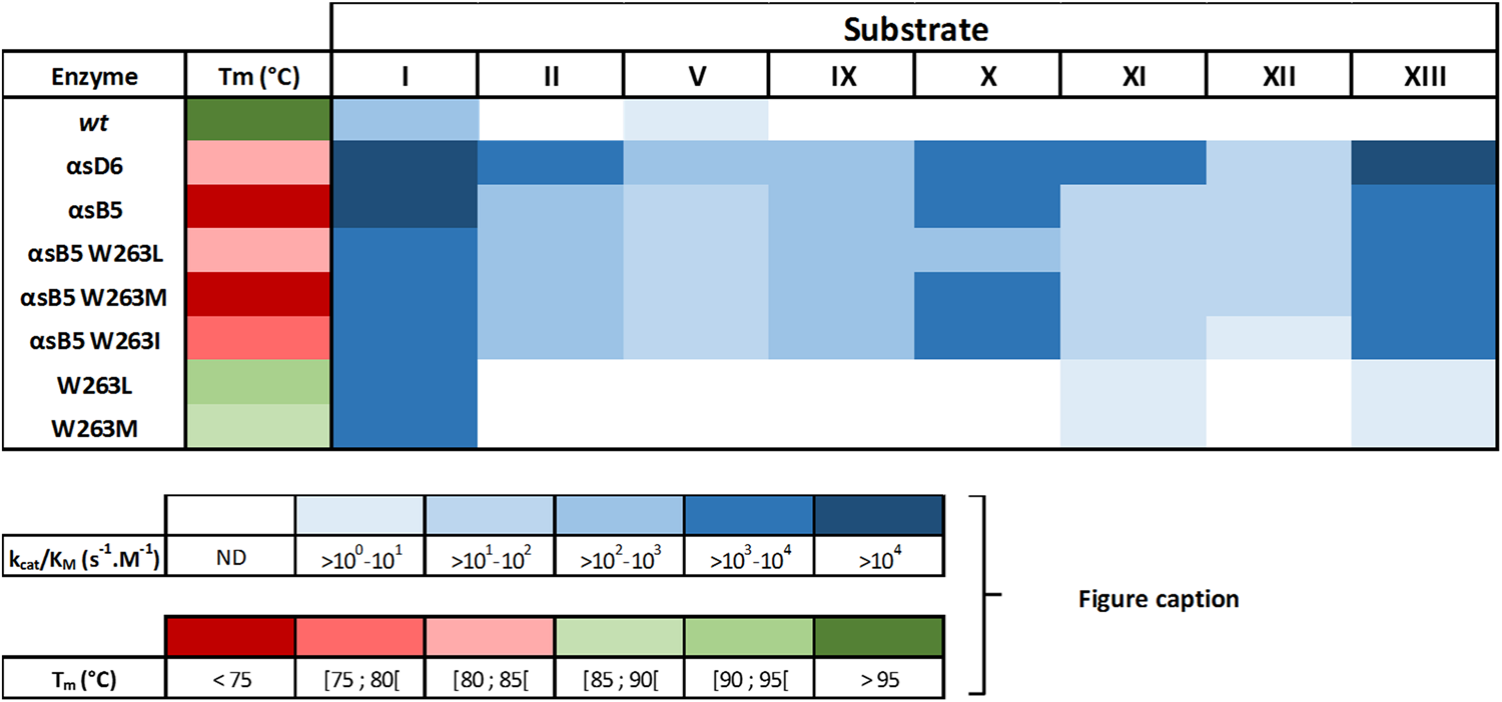
Overview of *Sso*Pox wild-type and variants properties. Catalytic efficiencies towards organophosphate-based pesticides are presented using a color code ranging from white to dark blue. Melting temperatures are presented using a color code ranging from red to green.

All mutants selected and used in this study are destabilized as compared to the *wt*-enzyme (Fig. 7; Table S4). Structures reveal that these mutations, located on loop 8, contribute to increasing the mobility of the loop. Interestingly, the ability of the tested mutants to hydrolyze a wide range of phosphotriesters, including compounds that were inhibitors for the *wt*-enzyme, correlates with a lower T_m_ (Fig. 7 **)**. This correlation suggest that the active site loop 8 degree of disorder may modulate the enzyme’s ability to bind and hydrolyze a variety of phosphotriesters, a wider conformational ensemble of loop 8 being correlated with a broader enzyme specificity. This observation is consistent with previous studies highlighting the importance of the destabilization of active site loops to evolve new activities and new substrate specificity^54,56^. Such flexible active site loops involved in enzymatic specificity supports the notion of fold polarity: a portion of the active site (e.g. the loop) is weakly connected to the enzyme scaffold and thereby makes it possible for new functions to evolve with few changes^57^.

### Mutant αsD6 is an active, broad spectrum phosphotriester biodecontaminant

Mutant αsD6 was further investigated for bioremediation considerations. Organophosphate-based pesticides are usually spread with concentrations in the millimolar range leading to micromolar-ranged contaminations. We thus investigated the potential of αsD6 for decontaminating pesticide solutions at 250 µM to simulate a contamination of groundwater or runoff waters. We investigated two $\text{[E]/}[S]$ ratios, 10^−2^ and 10^−3^ respectively, for decontaminating OP solutions and determined the time required to hydrolyze 95% of pesticide preparations (Fig. 3B; Figure S4). When considering the $\text{[E]/}[S]$ ratio of 10^−3^, 95% degradation was achieved for four substrates (ethyl-paraoxon, diazinon, fenitrothion and coumaphos) within 10 minutes. Two substrates were decontaminated within an hour (ethyl-parathion and chlorpyrifos) and fensulfothion was degraded in three hours.

When increasing enzyme concentration 10-fold ($\text{[E]/}[S]$ ratio of 10^−2^), five substrates (ethyl-paraoxon, ethyl-parathion, diazinon, fenitrothion and coumaphos) were decontaminated within two minutes, while fensulfothion needed ~20 minutes and the two other tested (chlorpyrifos and malathion) required 40 minutes. Given both its catalytic proficiency and high thermal stability (T_m_ = 82.5 °C), αsD6 is a promising candidate for organophosphorus compound bioremediation and, in particular, the decontamination of water runoffs, soils, food products and materials. Moreover, *Sso*Pox variants were previously shown to be compatible with immobilization steps including alginate beads and polyurethane-based coatings^48,58^, and could be of prime interest for the development of filtration devices for water treatment purposes.

## Methods

### Structure-based identification of mutations

Sharing the same (α/β)_8_ barrel topology, *Sso*Pox and *Bd*PTE structures (Pdb ID 2vc5 and 1dpm, respectively) were superimposed (Fig. 1) using PyMol^59^, making it possible to identify structurally equivalent residues at their respective active sites. In an effort to reshape the *Sso*Pox active site, side chains that were well superimposed between the two enzymes were mutated using Coot into the residue present in *Bd*PTE^60^, with the exception of V27 which was mutated in Ala (and not Gly) to avoid increased entropy (Fig. 1; Table S1). However, due to the major differences in loop 8 and 7 between PLLs and PTEs, numerous residues were not superimposable. These active site residues (Y99, L228, F229 and W263) were therefore mutated in an effort to mimic the *Bd*PTE active site cavity in terms of shape and chemical nature, as illustrated by the chimeric reconstruction of *Sso*Pox carrying all these mutations (Figure S1 **)**. Others mutations were also implemented in the dataset, e.g. C258A and C258L and different substitutions of the key position W263 (F/L/M/A) that have been shown to improve the phosphodiesterase activity^11^.

### Synthesis of the combinatorial library

The *Sso*Pox coding gene was amplified from the previously described pET22b-*Sso*Pox plasmid^46^. For each of the 14 mutations, a mutagenesis primer of 30–33 bp (~10–15 bp from each mutation side) was synthesized (Table S2). For close mutations, primers can share several mutations and different primers were synthesized and mixed to keep an equivalent statistical probability for each of the 14 mutations. Typically, 2 pmol of primer mixture and 100 ng of DNaseI (TaKaRa)-generated fragments of the *Sso*Pox gene were assembled as previously described^17^, followed by nested PCR with external cloning primers (*Sso*Pox-lib-pET-5′/3′) (Table S2). The PCR product was then cloned into a customized version of the pET32b(-ΔTrx) plasmid and then electroporated into *E*. *cloni* cells (Lucigen, USA). After agar-plate growth, plasmid extraction was performed to create the plasmid bank. Variant genes were PCR amplified using T7-prom and pET-RP primers (Table S2) and sequenced. The combinatorial library shows an average of 5.3 ± 3.3 library mutations by sequencing 10 randomly picked colonies, and 0.43 ± 0.53 random mutations per gene.

### Screening of the library

The plasmid library was used to transform the *Escherichia coli* strain BL21(DE_3_)-pGro7/GroEL (TaKaRa) to obtain colonies expressing a library of *Sso*Pox variants. Randomly picked clones (184), representing a coverage of 3.8% of the library, were grown on a 96-well plate in 500 µL of ZYP medium as previously described^11^. Production of proteins and chaperones was induced after five hours of culture at 37 °C by reducing the temperature to 25 °C, adding CoCl_2_ (0.2 mM) and adding arabinose (0.2%, w/v). After overnight growth, cell lysate was used to perform activity screening with 100 µM of paraoxon substrate (Figure S2B) after partial purification of the protein with a heating step of 15 minutes incubation at 70 °C^35,49^. The screening was performed in 50 mM HEPES pH 8, 150 mM NaCl, 0.2 mM CoCl_2_. Kinetics of paraoxon/methyl-parathion hydrolysis were monitored by following absorbance at 405 nm for 10 minutes using a microplate reader (Synergy HT, BioTek, USA) and the Gen5.1 software.

### Saturation library

Saturation mutagenesis directed on residue 263 of the αsB5 variant encoding gene, was performed using NNS degenerated primers (W263NNS direct: 5′-TGCACCATTGATNNSGGCACCGCAAAACCG-3′ and W263NNS reverse: 5′-CGGTTTTGCGGTGCCSNNATCAATGGTGCA-3′) and pET32b-ΔTrx carrying αsB5 coding gene as template. PCR amplification was performed with 2.5 U of PfuTurbo DNA polymerase (Agilent) according to the manufacturer’s recommendations [95 °C, 5 minutes; 20x (95 °C, 30 seconds; 55 °C, one minute; 68 °C, 15 minutes); 68 °C, 25 minutes]. DNA was digested with *Dpn*I to remove the methylated parental template. *E*. *coli* BL21(DE_3_)-pGro7/GroEL (TaKaRa) competent cells were transformed with the plasmid pool and plated on LB-agar supplemented with 100 µg.mL^−1^ ampicillin and 34 µg.mL^−1^ chloramphenicol. Over a theoretical diversity of 20 sequences, 181 variants were collected in a microplate containing LB (100 µg.mL^−1^ ampicillin and 34 µg.mL^−1^ chloramphenicol) for characterization. This oversampling by a factor of nearly 10-fold ensure a >99% probability to get the 20 possible substitutions.

Screening was performed as described above (Figure S2C). Clones were produced in liquid ZYP medium and their activity was screened with 100 µM of paraoxon substrate. The plasmids corresponding to the most interesting variants were extracted and the *Sso*Pox variant encoding genes were sequenced.

### Production-purification of *Sso*Pox wild-type and variants

The genes coding for the *Sso*Pox wild-type and its variant were cloned in a pET32b-Δtrx plasmid. Productions were performed using the *E*. *coli* BL21(DE_3_)-pGro7/GroEL (TaKaRa) chaperone expressing strain. Starter cultures were produced in an auto-inducible ZYP medium (supplemented with 100 µg.mL^−1^ ampicillin and 34 µg.mL^−1^ chloramphenicol). When OD_600nm_ reached a value of 0.8–1, CoCl_2_ was added (final concentration 0.2 mM) as well as L-arabinose (final concentration 2 g.L^−1^) to induce the production of chaperones GroEL/ES and the temperature was decreased to 23 °C for 16–20 hours. Cells were harvested by centrifugation (4400 g, 4 °C, 20 minutes) and resuspended in *lysis buffer* (50 mM HEPES pH 8.0, 150 mM NaCl, 0.2 mM CoCl_2_, 0.25 mg.mL^−1^ lysozyme, 0.1 mM PMSF and 10 µg.mL^−1^ DNAseI) and were stored at -80 °C. Cells were thawed and lysed in three steps of 30 seconds of sonication (Qsonica, Q700; Amplitude 45). Cell debris was removed by centrifugation (20000 g, 4 °C, 15 minutes). As *Sso*Pox and its variants are hyperthermostable, a pre-purification step was performed by heating the lysate for 30 minutes at 80 °C. Precipitated host proteins were removed by centrifugation (20000 g, 4 °C, 15 minutes). *Sso*Pox and its variants were collected by ammonium sulfate precipitation (75%) and resuspended in 8 ml of *activity buffer* (50 mM HEPES pH 8.0, 150 mM NaCl, 0.2 mM CoCl_2_). The remaining ammonium sulfate was eliminated by injection on a desalting column (HiPrep 26/10 desalting, GE Healthcare; ÄKTA Avant) and concentrated to 2 mL for separation on exclusion size chromatography (HiLoad 16/600 Superdex^TM^ 75 pg, GE Healthcare; ÄKTA Avant). Final purity was monitored by SDS-PAGE and protein concentration was measured with a NanoDrop 2000 spectrophotometer (Thermo Scientific).

### Kinetic assays

*Generalities*. Catalytic parameters were evaluated in triplicate at 25 °C, and recorded using a microplate reader (Synergy HT, BioTek, USA) and the Gen5.1 software, in a 6.2 mm path length cell for 200 µL reaction in 96-well plate, as previously explained^30^. Catalytic parameters were obtained by fitting the data to the Michaelis-Menten (MM) equation using Graph-Pad Prism 6 software. When V_max_ could not be reached in the experiments, catalytic efficiency was obtained by fitting the linear part of MM plot to a linear regression using Graph-Pad Prism 6 software. For some *Sso*Pox variants, the MM plot was fitted to the substrate inhibition equation using Graph-Pad Prism 6 software enabling us to determine a K_I_ for several substrates. Consequently, the calculated catalytic efficiencies in these conditions are true only at low substrate concentrations. In some other cases, saturation could not be reached, therefore ${k}_{{cat}}/{K}_{M}$ values were determined using linear regression. For Coumaphos, data were fitted to one-phase decay non-linear regression.

#### Phosphotriesterase activity characterization

The kinetic assays were carried out in *activity buffer*. The conditions used for pesticides were as follows: ethyl-paraoxon (**I**), ethyl-parathion (**II**), methyl-paraoxon (**III**) and methyl-parathion (**IV**) hydrolysis was recorded at 405 nm (ε = 17000 M^−1^.cm^−1^). The concentration interval was between 0.05 and 2 mM from 100 mM stock solution in ethanol^46^. Malathion (**V**) hydrolysis was monitored at 412 nm, (ε = 13400 M^−1^.cm^−1^)^46^. The concentration interval was between 0.05 and 2 mM from 100 mM stock solution in DMSO. DTNB was added to the activity buffer. Chlorpyrifos (**VI**) hydrolysis was recorded at 310 nm (ε = 5562 M^−1^.cm^−1^)^51^. The concentration interval was between 0.05 and 2 mM from 100 mM stock solution in 1-propanol. Diazinon (**VII**) hydrolysis was recorded at 228 nm (ε = 3300 M^−1^.cm^−1^)^27^. The concentration interval was between 0.05 and 2 mM from 200 mM stock solution in ethanol. Fenitrothion (**VIII**) hydrolysis was recorded at 358 nm (ε = 18700 M^−1^.cm^−1^)^52^. The concentration interval was between 0.05 and 2 mM from 200 mM stock solution in ethanol. Fensulfothion (**IX**) hydrolysis was recorded at 284 nm (ε = 8000 M^−1^.cm^−1^)^27^. The concentration interval was between 0.05 and 2 mM from 200 mM stock solution in methanol. Coumaphos (**X**) hydrolysis was monitored by fluorescence of released chlorferon (excitation 360/40 and emission 460/40)^51^. A linear correlation between fluorescence and chlorferon was found (Figure S3). Measurements were taken at 5 µM from a 200 mM stock solution in DMSO. We assumed that ${K}_{M}\gg [S]$, thus enabling us to estimate ${k}_{{cat}}/{K}_{M}$.

#### Lactonase activity characterization

Lactonase kinetics were performed using a previously described protocol^30^. The time course hydrolysis of lactones were performed in *lac buffer* (2.5 mM Bicine pH 8.3, 150 mM NaCl, 0.2 mM CoCl_2_, 0.25 mM Cresol purple and 0.5% DMSO) over a concentration range 0–2 mM for 3-oxo-C10 AHLs (**XI**) or 0–5 mM for undecanoic- γ/δ -lactones (**XII, XIII**). Cresol purple (pK_a_ 8.3 at 25 °C) is a pH indicator used to follow lactone ring hydrolysis by acidification of the medium. Molar coefficient extinction at 577 nm was evaluated recording absorbance of the buffer over an acetic acid range of concentration 0–0.35 mM.

### Bioremediation of pesticide solutions at 250 µM

For all OP substrates but malathion, experiments were performed in triplicate at 25 °C using a microplate reader (Synergy HT, BioTek, USA). Wavelengths were chosen as described in the kinetic assays section. Degradation of malathion was followed by GC/MS in triplicate. OP concentration was fixed at 250 µM. All the substrates were soluble at this concentration. The best variants of *Sso*Pox for each substrate were used, namely αsD6 for all the OPs. Two enzyme-to-substrate ratios $\text{[E]/}[S]$ were used, 10^−2^ and 10^−3^, corresponding to enzyme concentrations of 90 µg.ml^−1^ and 9 µg.ml^−1^ respectively. The reactions were monitored until the plateau was reached. Experimental measures were obtained using Gen5.1 software, then analyzed with GraphPad Prism 6 software. Curves were then fitted using One-Phase Decay non-linear regression with the equation (1):

1 $$Y=(Y0-Plateau)\times {e}^{(-kt)}+Plateau$$

where *Y*0 = 0% and *Plateau* = 100%. The rate constant *k* was determined and the time required to observe a 95% decontamination was calculated accordingly. The curves and results of the fits are shown in Figure S4.

Degradation at 95% was confirmed using a final point by GC/MS for all pesticides with a 10^−3^ enzyme-to-substrate ratio. 100 µL of *activity buffer* solution was first extracted with 100 µL chloroform. Organic extracts were analyzed by using a Clarus 500 gas chromatograph equipped with a SQ8S MS detector (Perkin Elmer, Courtaboeuf, France). 1 µL of organic extract was volatilized at 220 °C (split 15 mL.min^−1^) in a deactivated FocusLiner with quartz wool (SGE, Ringwood, Australia) and compounds separated on an Elite-5MS column (30 m, 0.25 mm i.d., 0.25 mm film thickness) for 12 minutes using a temperature gradient (80–280 °C at 30 °C.min^−1^, five minutes’ hold). Helium flowing at 2 mL.min^−1^ was used as the carrier gas. The MS inlet line was set at 280 °C and the electron ionization source at 280 °C and 70 eV. Full scan monitoring was performed from 40 to 400 *m/z* in order to identify chemicals by spectral database search using MS Search 2.0 operated with the Standard Reference Database 1 A (National Institute of Standards and Technology, Gaithersburg, MD, USA). *m/z* is the mass-to-charge ratio of the base peak fragment detected for each molecule. In the case of weak pesticide signals, extracted ion chromatograms were generated with base peak ions to confirm the presence of chemicals (Paraoxon (**I**) and Parathion ethyl (**II**) *m/z* 109; Malathion (**V**) and Fenitrothion (**VIII**) *m/z* 125; Diazinon (**VII**) *m/z* 137; Coumaphos (**X**) *m/z* 97; Fensulfothion (**IX**) *m/z* 293; and Chlorpyrifos (**VI**) *m/z* 197). Selected Ion Recording using base peaks ions was applied in order to specifically monitor pesticides and collect peak areas for kinetics. All samples were analyzed over short periods of time to avoid signal drift. All data were processed using Turbomass 6.1 (Perkin Elmer).

### Crystallization

Crystallization assays were performed as previously described^42,46^. Crystallization was performed using the hanging drop vapor diffusion method in 96-well plates (Greiner Microplate, 96 well, PS, F-bottom) on ViewDrop II seals (TPP Labtech). Equal volumes (0.5 µL) of protein and reservoir solutions were mixed, and the resulting drops were equilibrated against a 150 µL reservoir solution containing 20–30% (w/v) PEG 8000 and 50 mM Tris-HCl buffer (pH 8). Crystals appeared after few days at 4 °C.

### Data collection and structure refinement

Crystals were first transferred to a cryoprotectant solution consisting of the reservoir solution and 20% (v/v) glycerol. Crystals were then flash-cooled in liquid nitrogen. X-ray diffraction data were collected at 100 K using synchrotron radiation at the ID23-1 beam line (ESRF, Grenoble, France) and using an ADSC Q315r detector. X-ray diffraction data were integrated and scaled with the *XDS* package (Table 1)^61^. The phases were obtained using the native structure of *Sso*Pox (PDB code 2vc5) as a starting model, performing a molecular replacement with *MOLREP* or *PHASER* ^62,63^. The models were built with *Coot* and refined using *REFMAC* ^60,64^. We note that three structures presented in this work exhibit one disordered monomer (and low corresponding electronic density): αsB5 (monomer D, total of 4 monomers), αsD6 (monomer C, out of a total of 4 monomers) and αsC6 (monomer C, total of 4 monomers). Structure illustrations were performed using *PyMOL* ^48^.

### Relative B-factor analysis

The occupancies of all residues in all tested structures were set to 1 for this analysis. For residues with alternate conformations, the sums of occupancies were set to 1. Structures were re-refined with *REFMAC* ^60,64^. The relative B-factor values were obtained by normalizing the B-factor values of each residue by the average B-factor of the whole structure as previously described^11,54,57^.

## Electronic supplementary material

Supplementary Information
