# Supplementary material for: Rational engineering of a native hyperthermostable lactonase into a broad spectrum phosphotriesterase
Source: Sci Rep. 2017 Dec 1;7:16745. doi: 10.1038/s41598-017-16841-0 (PMC5711954; doi:10.1038/s41598-017-16841-0)
Supplement: Supplementary file 1 — Supplementary Information [file 41598_2017_16841_MOESM1_ESM.pdf]

**SUPPLEMENTARY DATA**

# Rational engineering of a native hyperthermostable lactonase into a broad spectrum phosphotriesterase

Pauline Jacquet[1°], Julien Hiblot[1‡°], David Daudé[2°], Céline Bergonzi[1,3], Guillaume Gotthard[1], Nicholas Armstrong[1], Eric Chabrière[1#], Mikael Elias[3#]

1 - CNRS UMR 7278, IRD198, INSERM U1095, APHM, Institut Hospitalier Universitaire Méditerranée-Infection, Aix-Marseille Université, 19-21 Bd Jean Moulin 13005 Marseille, France.

2 - Gene&GreenTK, IHU Méditerranée Infection, 19-21 Bd Jean Moulin, 13005 Marseille, France

3 - University of Minnesota, Department of Biochemistry, Molecular Biology and Biophysics & Biotechnology Institute, St. Paul, MN 55108, USA

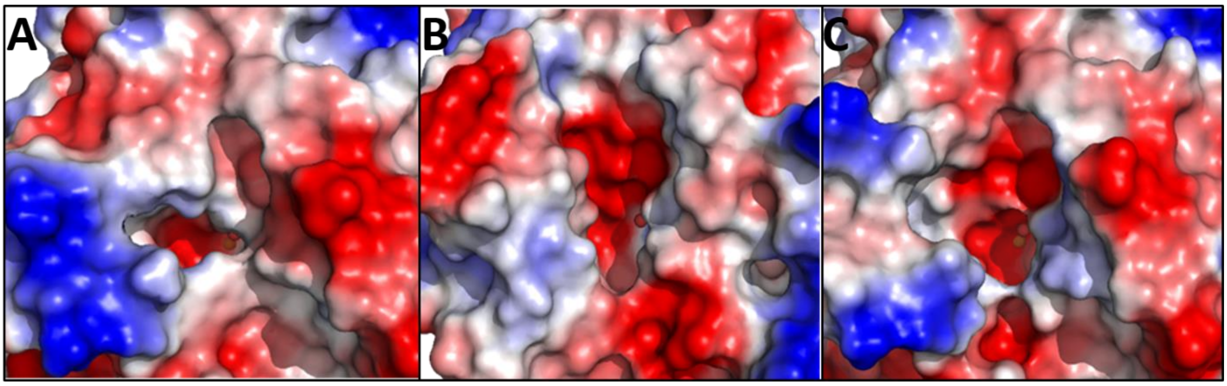


**Figure S1: Structure-based active site cavity redesign.** Active site cavity view of (**A**) *wt* *Sso*Pox, (**B**) *Bd*PTE and (**C**) *Sso*Pox modelled with all 14 designed mutations (Table S1). The mutations modelled were constructed *in silico* and energy was minimized using *Swiss PDB viewer*.[59] Electrostatic potentials were calculated using PyMOL[60] and are shown on the molecular surface. Electrostatic potentials are represented at the enzyme's surfaces. Blue, red and white indicates positively-, negatively-, and neutrally-charged areas, respectively.

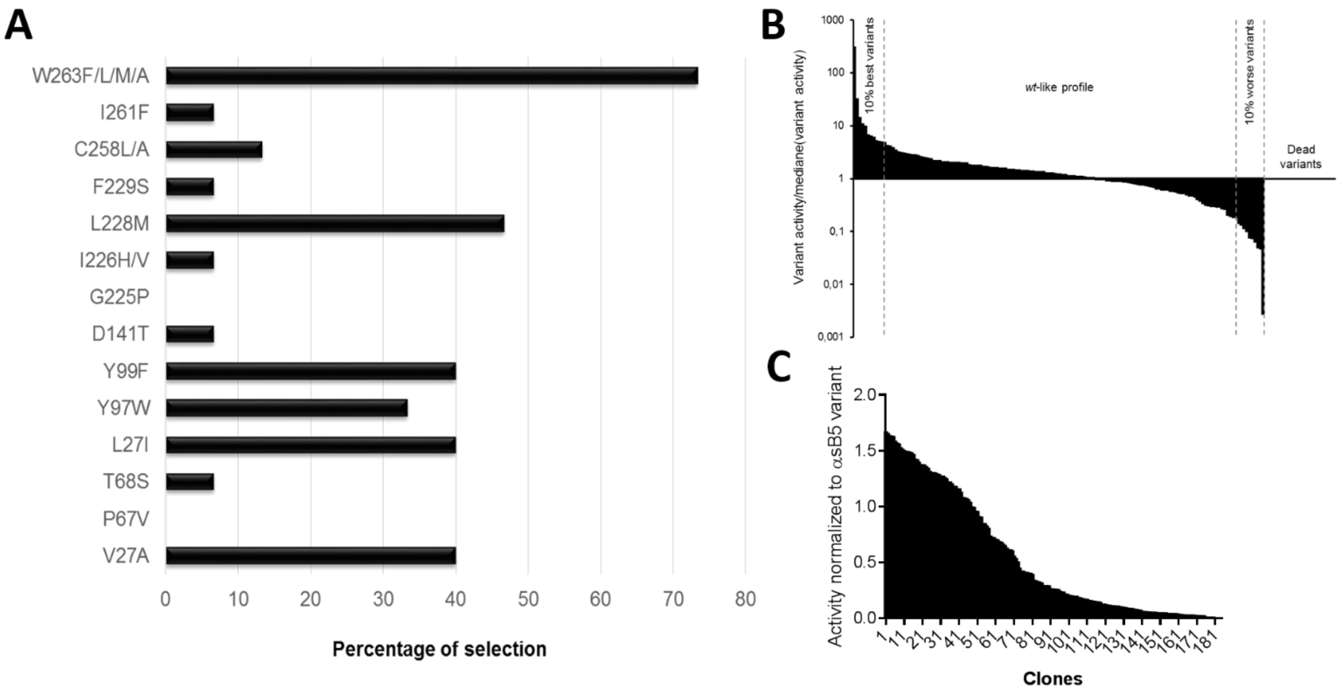


**<u>Figure S2: Library screening data</u>.** (**A**) Occurrence of mutations in the 14 best sequenced clones. (**B**) Screening profile of the combinatorial mutation library (184 clones) with paraoxon as a substrate. Activities ($mOD_{405nm}$ $min^{-1}$) were normalized to the median value. (**C**) Screening of the position 263 saturation library (αsB5 background) with paraoxon as the substrate. Activity was normalized to αsB5 paraoxonase activity.

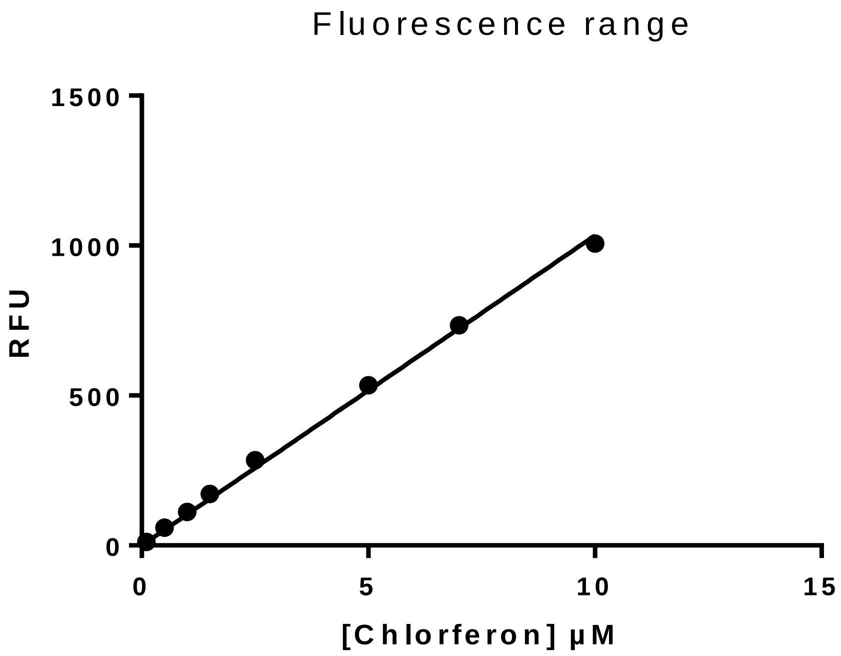


| Best-fit values | |
|---|---|
| Slope | 103,3 ± 0,6551 |
| Equation | Y = 103,3*X - 0,0 |
| $R^2$ | 0.9975 |

**Figure S3. Chlorferon vs Fluorescence (360 (40) / 460 (40)) range of linearity**

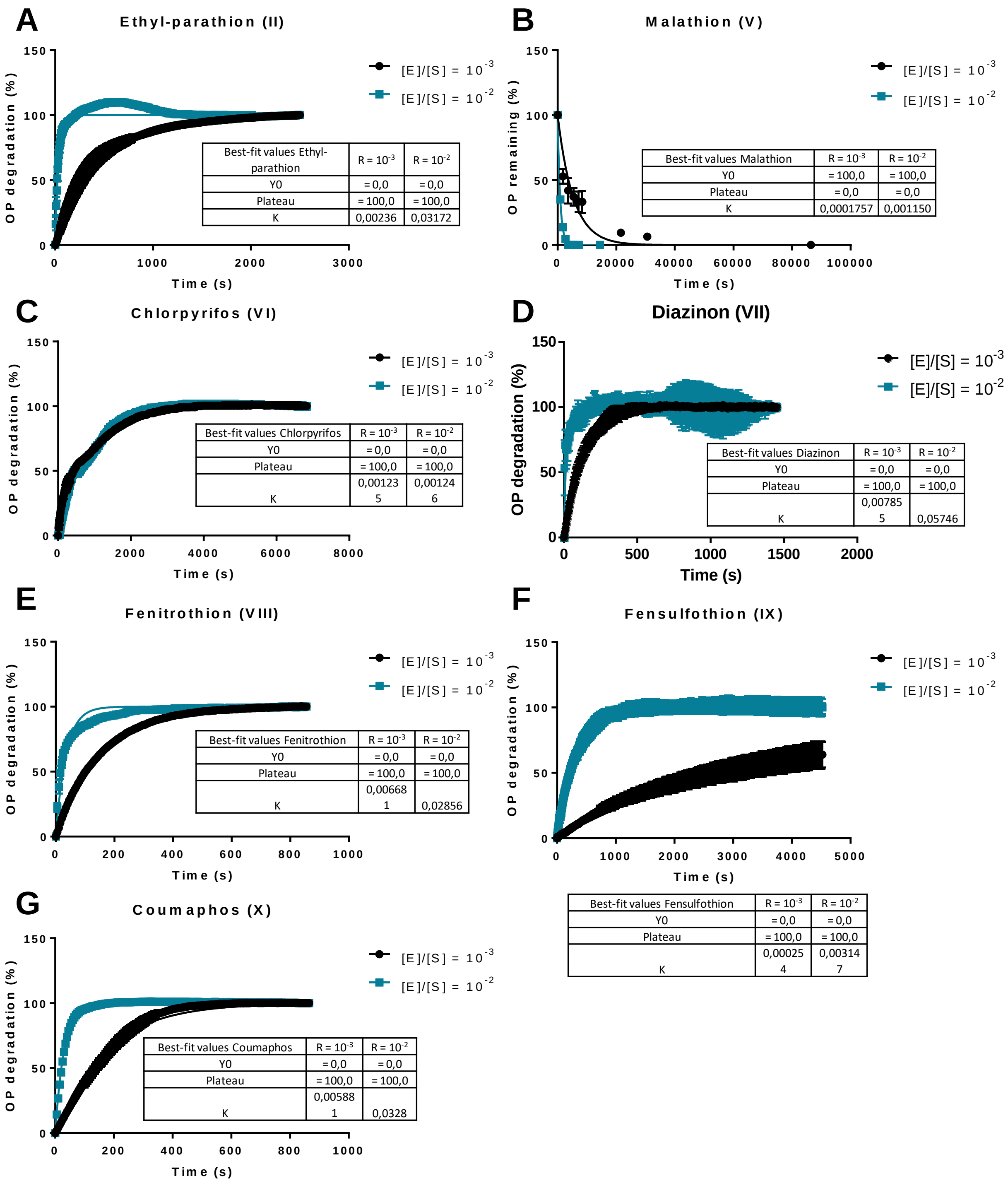


**Figure S4. Bioremediation of organophosphorus insecticides (250 µM)**

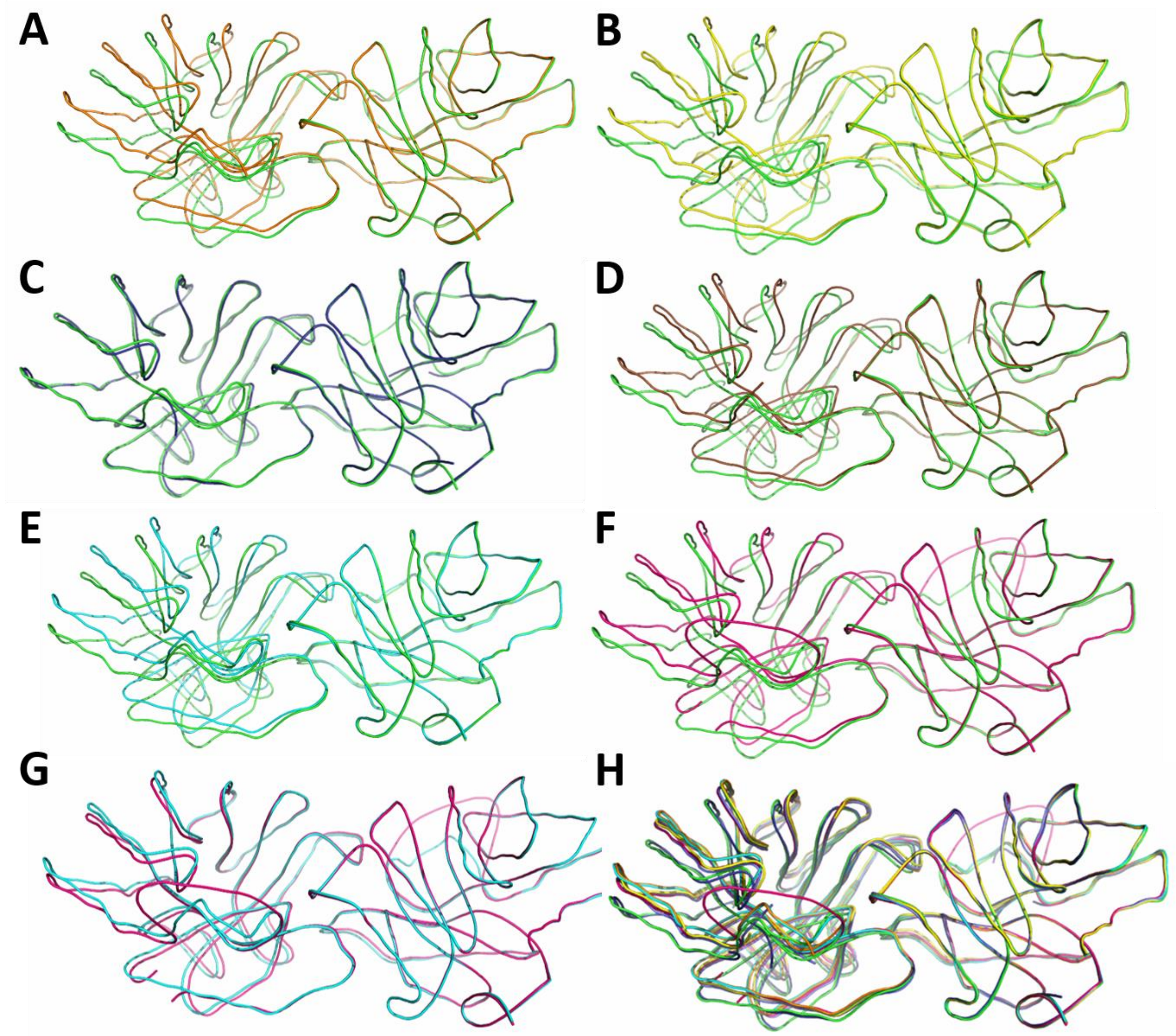


**Figure S5. Structural superposition of wt-*Sso*Pox and mutants dimers**. The monomers on the right were superimposed, and the difference with the left monomers indicates the relative reorientation of dimerized monomers. (**A**) Superposition of wt-*Sso*Pox (green) and αsA1 (orange). (**B**) Superposition of wt-*Sso*Pox (green) and αsB5 (yellow). (**C**) Superposition of wt-*Sso*Pox (green) and αsC6 (blue). (**D**) Superposition of wt-*Sso*Pox (green) and αsD6 (brown). (**E**) Superposition of wt-*Sso*Pox (green) and αsA6-CC (cyan). (**F**) Superposition of wt-*Sso*Pox (green) and αsA6-OC (magenta). (**G**) Superposition of αsA6-OC (magenta) and αsA6-CC (cyan). (**H**) Superposition of wt-*Sso*Pox (green), αsA1 (orange), αsB5 (yellow), αsD6 (brown), αsC6 (blue), αsA6-OC (magenta) and αsA6-CC (cyan).

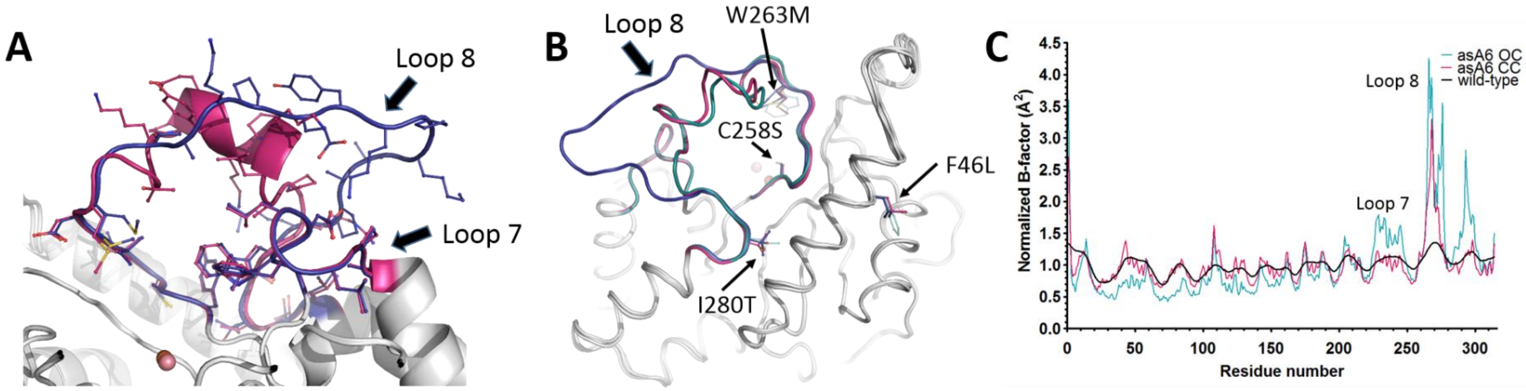


**<u>Figure S6. Mutant αsA6 loop-8 conformational flexibility.</u>** (**A**) Superposition of the open conformation (OC, blue) and the closed conformation (CC, magenta), of loop 8 in two distinct mutant αsA6 crystal structures. (**B**) Location of the four mutations of mutant αsA6 in the structure. The wt *Sso*Pox, the CC, and the OC loop 8 conformations are shown in green, magenta and blue, respectively. We note that I280T and C258S are located at the very beginning and end of loop 8. (**C**) Positional distributions of normalized B-factor values (the x-axis represents residue number) for wt-*Sso*Pox (black line), αsA6-OC (blue line) and (magenta line).

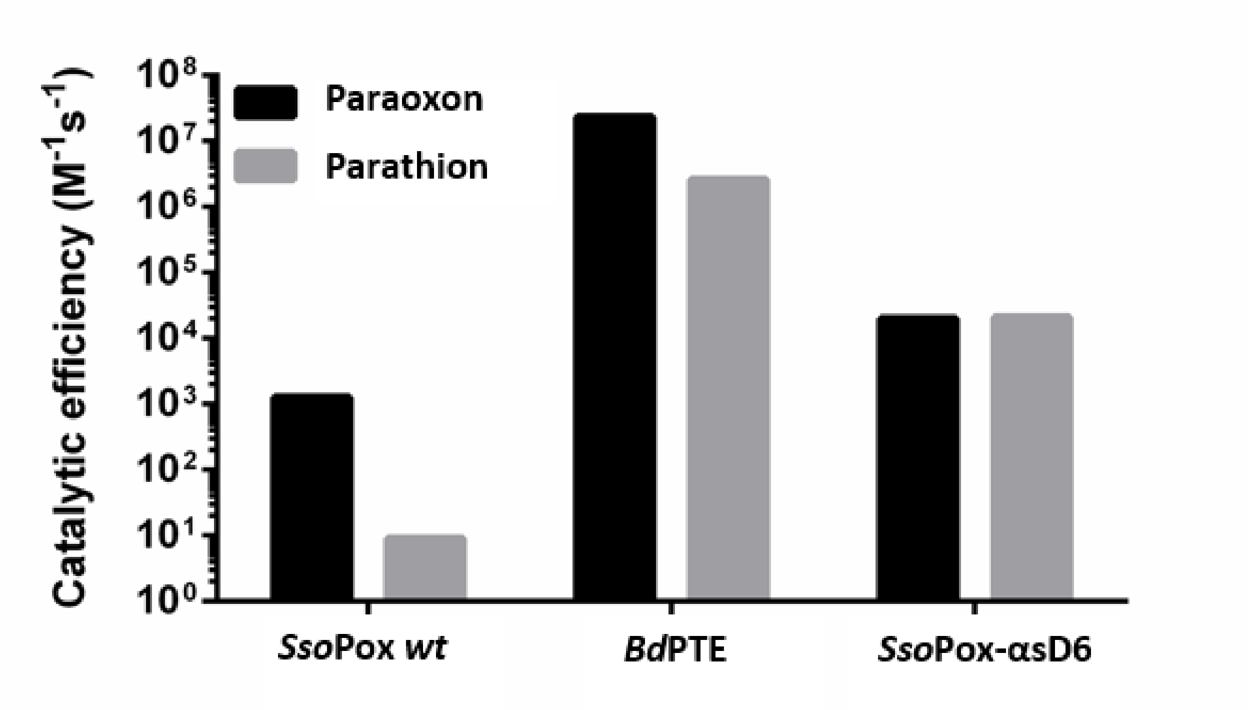


**Figure S7: Thiono-effect in *Sso*Pox.** Catalytic efficiencies of enzymes, *Sso*Pox, *Bd*PTE and *Sso*Pox-αsD6 with methyl-paraoxon (black) and methyl-parathion (gray) as substrates are shown.

# Table S1: Mutation database

| | *Sso*Pox posistion | Corresponding position in *Bd*PTE | Redesign | Additional mutations | Mutations in database |
|---|---|---|---|---|---|
| First set of mutations | V27 | G60 | - | - | V27A |
| | P67 | V101 | - | - | P67V |
| | T68 | S102 | - | - | T68S |
| | L72 | I106 | - | - | L72I |
| | Y97 | W131 | - | - | Y97W |
| | D141 | T173 | - | - | D141T |
| | G225 | P256 | - | - | G225P |
| | L226 | H257 | - | - | L226H |
| | C258 | L303 | - | A | C258/A-L |
| | I261 | F306 | - | - | I261F |
| Second set of mutations | Y99 | - | F | - | Y99F |
| | L228 | - | M | - | L228M |
| | F229 | - | S | - | F229S |
| | W263 | - | A | Saturation | W263/All |

# Table S2: Oligonucleotide sequences used in this study

| Primer name | | | Primer sequence |
|---|---|---|---|
| Clonage | T7-prom | | TAA TAC GAC TCA CTA TAG GG |
| | pET-RP | | GCT AGT TAT TGC TCA GCG G |
| | SsoPox-lib-pET-5' | | CAT GCC ATG GCG CGC ATT CCG CTG GTT GGT AAA G |
| | SsoPox-lib-pET-3' | | AAG GAA AAA AGC GGC CGC TTA TTA GCT AAA GAA TTT TTT CGG ATT TTC |
| **Primer name** | | **Encoding variation** | **Primer sequence** |
| Primers used in ISOR protocol | SP1 | V27A | GAA CAT CTG CGT **GCA** TTT AGC GAA GCA GTT |
| | SP2 | P67V-T68S | AAA ACC ATT GTT GAT **GTG AGT** GTT ATG GGT |
| | SP3 | P67V | AAA ACC ATT GTT GAT **GTG** ACC GTT ATG GGT |
| | SP4 | T68S | AAA ACC ATT GTT GAT CCG **AGT** GTT ATG GGT |
| | SP5 | L72I | GTT ATG GGT **ATT** GGT CGT GAT ATT CGT TTT |
| | SP6 | Y97W-Y99F | GGC ACC GGT ATT **TGG** ATT **TTT** ATC GAT CTG CCG |
| | SP7 | Y97W | GGC ACC GGT ATT **TGG** ATT TAT ATC GAT CTG CCG |
| | SP8 | Y99F | GGC ACC GGT ATT TAT ATT **TTT** ATC GAT CTG CCG |
| | SP9 | D141T | AAA ATT GCA GCC **ACC** GAA CCG GGT ATT ACC |
| | SP10 | L226H-228L/M-229F/S | GAT CGT TAT GGT **CAC** GAC **MTG TYT** CTG CCG GTT |
| | SP11 | L226V-228L/M-229F/S | GAT CGT TAT GGT **GTG** GAC **MTG TYT** CTG CCG GTT |
| | SP12 | G225P-228L/M-229F/S | GAT CGT TAT **CCG** CTG GAC **MTG TYT** CTG CCG GTT |
| | SP13 | G225P-L226H-228L/M-229F/S | GAT CGT TAT **CCG CAC** GAC **MTG TYT** CTG CCG GTT |
| | SP14 | G225P-L226V-228L/M-229F/S | GAT CGT TAT **CCG GTG** GAC **MTG TYT** CTG CCG GTT |
| | SP15 | 228L/M-229F/S | GAT CGT TAT GGT CTG GAC **MTG TYT** CTG CCG GTT |
| | SP16 | C258L | ATT AGC CAT GAT TAT **CTG** TGC ACC ATT GAT |
| | SP17 | C258A | ATT AGC CAT GAT TAT **GCC** TGC ACC ATT GAT |
| | SP18 | I261F | GAT TAT TGC TGC ACC **TTT** GAT TGG GGC ACC |
| | SP19 | W263F | TGC ACC ATT GAT **TTT** GGC ACC GCA AAA CCG |
| | SP20 | W263M | TGC ACC ATT GAT **ATG** GGC ACC GCA AAA CCG |
| | SP21 | W263L | TGC ACC ATT GAT **CTG** GGC ACC GCA AAA CCG |
| | SP22 | W263A | TGC ACC ATT GAT **GCG** GGC ACC GCA AAA CCG |

## Table S3: Selected positions for phosphotriesterase activity improvement

| Position | 27 | 67 | 68 | 72 | 97 | 99 | 141 | 225 | 226 | 228 | 229 | 258 | 261 | 263 | Additional | Total variations |
|---|---|---|---|---|---|---|---|---|---|---|---|---|---|---|---|---|
| **wt** | V | P | T | L | Y | Y | D | G | I | L | F | C | I | W | | |
| **B3** | A | | | I | W | | | | | M | | | | | | V27A/L72I/Y97W/L228M |
| **F2** | | | | | | | | | | M | | | | L | | L228M/W263L |
| **F8** | | | | I | | | | | | | | | | M | K54I | K54I/L72I/W263M |
| **B2** | A | | | I | | F | T | | | M | | | | M | G7S/V28A | G7S/V27A/V83A/L72I/Y99F/D141T/L228M/W263M |
| **H8** | | | S | | W | F | | | | M | | | | | | T68S/Y97W/Y99F/L228M/ |
| **E3** | A | | | I | | | | | | | | | | M | V83A/E173T | V27A/L72I/V83A/E173T/W263M |
| **A4** | | | | | | F | | | | M | | | | M | G193S | Y99F/G193S/L228M/W263M |
| **A1** | | | | | | | | | | | | L | F | A | | C258L/I261F/W263A |
| **A6** | | | | | | | | | | | | A | | M | F46L/I280T | F46L/C258A/W263M/I280T |
| **B5** | A | | | | W | F | | | V | | | | | | I76T/L130P | V27A/I76T/Y97W/Y99F/L130P/L226V |
| **C6** | | | | I | | F | | | | M | S | | | L | I122L | L72I/Y99F/I122L/L228M/F229S/W263L |
| **D6** | A | | | | W | | | | | M | | | | M | | V27A/Y97W/L228M/W263M |
| **D1** | | | | | W | | | | | | | | | | | Y97W |
| **D5** | | | | I | | | | | | | | | | F | | L72I/W263F |
| **A5** | A | | | | | F | | | | | | | | L | | V27A/L72I/Y99F/W263L |
| **Incorporation** | 6 | 0 | 1 | 6 | 5 | 6 | 1 | 0 | 1 | 7 | 1 | 2 | 1 | 11 | | |
| **Ratio** | 0.4 | 0 | 0.07 | 0.4 | 0.33 | 0.4 | 0.07 | 0 | 0.07 | 0.47 | 0.07 | 0.13 | 0.07 | 0.73 | | |

# Table S4: Tm values of *Sso*Pox variants

| Enzyme | Mutations | $T_m$ (°C) |
|---|---|---|
| ***wt*** | * | 106 |
| **αsD6** | V27A/Y97W/L228M/W263M | 82.5 ± 1,8 |
| **αsB5** | V27A/I76T/Y97W/Y99F/L130P/L226V | 70.4 ± 3,1 |
| **αsB5 W263M** | V27A/I76T/Y97W/Y99F/L130P/L226V/W263M | 69.1 ± 2,3 |
| **αsB5 W263L** | V27A/I76T/Y97W/Y99F/L130P/L226V/W263L | 80.2 ± 4,0 |
| **αsB5 W263I** | V27A/I76T/Y97W/Y99F/L130P/L226V/W263I | 77.9 ± 1,3 |
| **W263M** | W263M | 85.3 ± 0,9 |
| **W263L** | W263L | 92.0 ± 2,1 |

**Melting temperature determination.** Circular Dichroism (CD) spectra were obtained using a Jasco J-815 CD spectrometer with a Pelletier-type temperature control system (Jasco MPTC-490S) in a 1 mm thick quartz Starna® cell and using Spectra Manager software. Experiments were carried out in 10 mM phosphate buffer at pH 8.0. Proteins concentrations were within 0.2–0.4 mg.mL$^{-1}$, denaturation was followed at 222 nm with temperature increased from 25 to 85°C (with 5°C.min$^{-1}$ increment). A range of between 0.5 and 3 M of guanidinium chloride in 10 mM phosphate buffer at pH 8.0 was applied. Data were analyzed with GraphPad Prism 6 using Boltzmann sigmoidal equation, and $T_m$ at 0 M of guanidinium chloride was then extrapolated by linear regression.

**Thermal stability of the engineered variants**

We measured the melting temperature of variants αsD6, αsB5, αsB5-W263L, αsB5-W263M and αsB5-W263I in this study while the $T_m$ values of *wt*, W263M and W263L were previously reported (**Table S4**).[7] All variants showed decreased $T_m$ values as compared to the wild-type enzyme. These values ranged from 69 °C to 92 °C for αsB5-W263M and W263L, respectively. As previously reported, the role of residue 263 is crucial for overall enzyme stability. The stability of mono-substituted variants was globally less affected than the αsD6 and αsB5 scaffolds. The latter's Tm value decreased by 36 °C as compared to *Sso*Pox-WT, while the addition of a mutation at position W263 to its background was not destabilizing, suggesting that their contribution to stability may be different. While the $T_m$ values for the best variants have decreased, they remain very stable (e.g. αsD6 has a $T_m$ of 82.5°C), as compared with their mesophilic counterparts ($T_{m,BdPTE}$=67°C).[61] Additionally, the expression level of the *Sso*Pox and its mutants were ≈100-150 mg.l$^{-1}_{culture}$, a higher level than the *Bd*PTE ( ≈3 mg.l$^{-1}_{culture}$)and ≈50 mg.l$^{-1}_{culture}$ for the improved *Bd*PTE-S5 variant.[61]

Interestingly, the range of recognized substrate was broadened when thermostability decreased which is consistent with previous reports suggesting that mutations often increase activity at the cost of stability (**Figure 7**).[62,63]
